# targetTB: A target identification pipeline for *Mycobacterium tuberculosis *through an interactome, reactome and genome-scale structural analysis

**DOI:** 10.1186/1752-0509-2-109

**Published:** 2008-12-19

**Authors:** Karthik Raman, Kalidas Yeturu, Nagasuma Chandra

**Affiliations:** 1 Supercomputer Education and Research Centre and Bioinformatics Centre, Indian Institute of Science, Bangalore 560 012, INDIA

## Abstract

**Background:**

Tuberculosis still remains one of the largest killer infectious diseases, warranting the identification of newer targets and drugs. Identification and validation of appropriate targets for designing drugs are critical steps in drug discovery, which are at present major bottle-necks. A majority of drugs in current clinical use for many diseases have been designed without the knowledge of the targets, perhaps because standard methodologies to identify such targets in a high-throughput fashion do not really exist. With different kinds of 'omics' data that are now available, computational approaches can be powerful means of obtaining short-lists of possible targets for further experimental validation.

**Results:**

We report a comprehensive *in silico *target identification pipeline, targetTB, for *Mycobacterium tuberculosis*. The pipeline incorporates a network analysis of the protein-protein interactome, a flux balance analysis of the reactome, experimentally derived phenotype essentiality data, sequence analyses and a structural assessment of targetability, using novel algorithms recently developed by us. Using flux balance analysis and network analysis, proteins critical for survival of *M. tuberculosis *are first identified, followed by comparative genomics with the host, finally incorporating a novel structural analysis of the binding sites to assess the feasibility of a protein as a target. Further analyses include correlation with expression data and non-similarity to gut flora proteins as well as 'anti-targets' in the host, leading to the identification of 451 high-confidence targets. Through phylogenetic profiling against 228 pathogen genomes, shortlisted targets have been further explored to identify broad-spectrum antibiotic targets, while also identifying those specific to tuberculosis. Targets that address mycobacterial persistence and drug resistance mechanisms are also analysed.

**Conclusion:**

The pipeline developed provides rational schema for drug target identification that are likely to have high rates of success, which is expected to save enormous amounts of money, resources and time in the drug discovery process. A thorough comparison with previously suggested targets in the literature demonstrates the usefulness of the integrated approach used in our study, highlighting the importance of systems-level analyses in particular. The method has the potential to be used as a general strategy for target identification and validation and hence significantly impact most drug discovery programmes.

## Background

It is estimated that about two billion people, equalling one-third of the world's total population are infected with *M. tuberculosis *(*Mtb*) [1]. In 2006 alone, 1.7 million people died of tuberculosis (TB). TB is also the leading killer among HIV-infected people with weakened immune systems. The disease is also of particular interest to India and Asia, with more than half of all deaths occurring in Asia. Further, about 500,000 new multi-drug resistant TB cases are estimated to occur every year [1].

Currently, over 20 drugs are available for TB, of which, four of them, viz. isoniazid, rifampin, pyrazinamide and ethambutol are used as front-line drugs. Injectable drugs such as kanamycin, amikacin, capreomycin and viomycin are preferred next for treatment. Fluoroquinolones such as ciprofloxacin, ofloxacin have been found to be indispensable in the treatment of multi-drug resistant TB. Second-line bacteriostatics, such as *p*-aminosalicylic acid, ethionamide and cycloserine have established clinical efficacy but have more prominent side effects [2]. Isoniazid and ethionamide are inhibitors of mycolic acid synthesis [3,4], while cycloserine and ethambutol inhibit synthesis of peptidoglycan [5] and cell wall arabinogalactan [6,7] respectively, weakening the cell wall of the bacterium. Rifampin and Amikacin exert their pharmacological action by inhibiting bacterial RNA or protein synthesis [8-10]. As in the case of most other prescription drugs used currently, these were also discovered without the advantage of detailed molecular level information about the targets. A common strategy used in the past few decades for drug discovery involves finer structural optimisations, by starting with a lead compound that has already shown some success. Very often, this amounts to finding a newer improved drug, which modifies the function of the same target as the lead compound. This does not automatically lead to consideration of newer targets or even newer mechanisms of action. It is no surprise, therefore, that only a small fraction of the proteins in the bacterial genome have been explored as drug targets.

The existing drugs, although of immense value in controlling the disease to the extent that is being done today, have several shortcomings, the most important of them being the emergence of drug resistance rendering even the front-line drugs inactive. In addition, drugs such as rifampin have high levels of adverse effects making them prone for patient incompliance. Another important problem with most of the existing anti-mycobacterials, is their inability to act upon latent forms of the bacillus. In addition to these problems, the vicious interactions between the human immunodeficiency virus and TB have led to further challenges for anti-tubercular drug discovery [11]. For example, protease inhibitors have been shown to be incompatible with rifampin-containing anti-TB regimens [12]. As drug discovery efforts are increasingly becoming rational and much less dependent on trial and error, identification of appropriate targets becomes a fundamental pre-requisite.

Traditionally, targets have been identified through knowledge of the function of individual protein molecules, where their function has been well-characterised. Potential targets thus identified are generally taken through a validation process involving whole-cell or animal experiments, gene knock-outs or site-directed mutagenesis that lead to loss-of-function phenotypes. Target validation is one of the critical steps in drug discovery, where a lot of time and money is spent in the pharmaceutical industry. The need for systematic and large-scale validation in the post-genomic era has led to the usage of computational methods for validation [13]. Here, we seek to apply various *in silico *techniques for the identification and validation of drug targets, specifically for *Mtb*. *In silico *methods have the advantage of speed, low cost and even more importantly, provide a systems view of the whole microbe at a time, which enables asking questions that are often difficult to address experimentally. Drug discovery has witnessed a paradigm shift from the traditional medicinal chemistry-based ligand-oriented drug discovery approaches to rational drug target identification and target-driven lead discovery, by targeting the molecular mechanisms of disease. A number of studies have been carried out by various experimental methods to identify drug targets in *Mtb *[14]. Attempts have also been made for the same purpose, based on sequence comparisons of metabolic enzymes [15], and by using various features such as Lipinski druggability at the sequence level and metabolic choke-points at the systems-level [16].

Establishing systems biology concepts and understanding the microbe as a whole opens up new opportunities for computational target identification. Here, we report a comprehensive *in silico *target identification pipeline for *Mtb*, which can also be used as a general framework for *in silico *target identification. We focus our analysis at the systems level, based on network analyses and flux balance analyses (FBA), and further validating it based on sequence analyses and structural comparisons. We have used novel algorithms for the comparison of protein structures and identifying similarity of target pockets with pockets in the human proteome, which could initiate adverse drug effects. Gene expression data have also been considered to render the analysis more comprehensive.

## Methods

### The targetTB Pipeline

A new multi-level target identification pipeline, including a novel method for structural comparison of proteins has been developed. Different *levels of abstraction *are used for analysis, as discussed below. A summary of the several datasets used in these analyses is given in Table 1.

**Table 1:** Datasets used in this study

**Reference**	**Short Description**
Beste *et al *(2007) [20] (GSMN-TB)	Reports GSMN-TB, a genome-scale metabolic model of *Mtb*, consisting of 849 unique reactions and 739 metabolites, and involving 726 genes. *In silico *gene deletions have been performed, using FBA, identifying genes essential for growth.
Jamshidi *et al *(2007) [19] (*Mtb iNJ*661)	Reports the genome-scale metabolic reconstruction of the *in silico *strain *Mtb iNJ*661, comprising 661 genes and 939 reactions. *In silico *gene deletions have been performed, using FBA, identifying genes essential for growth, as well as slow-growing mutants.
Raman *et al *(2005) [21] (MAP)	Reports an FBA of the mycolic acid pathway (MAP) in *Mtb*, comprising 217 reactions involving 197 metabolites and mediated by 28 proteins. *In silico *gene deletions have been performed, detailing genes essential for mycolic acid biosynthesis.
Sassetti *et al *(2003) [23] (TraSH)	Reports the use of transposon site hybridisation (TraSH) mutagenesis to comprehensively identify the genes required by *Mtb *for optimal growth.
ModBase [25]	A database of structural models of proteins from various organisms, including *Mtb *and Human, based on homology modelling
Gao *et al *(2005) [30] (Gao-expression)	Reports the variability in gene expression patterns among ten clinical isolates of *Mtb*, as well as the laboratory strains H37Rv and H37Ra, growing in liquid culture.
Rachman *et al *(2006) [31] (Rxsachman-expression)	Reports genome-wide expression analysis of *Mtb *from clinical lung samples, as well as *in vitro*.
Boshoff *et al *(2004) [32] (Boshoff-expression)	Reports gene expression of *Mtb *in response to several drugs/inhibitors of metabolism, as well as under persistence, starvation and different pH/media.
Muttucumaru *et al *(2004) [40] (Muttucumaru-expression)	Reports global gene expression in aerobic, microaerophilic and anaerobic cultures.
Voskuil *et al *(2004) [39] (Voskuil-expression)	Reports a genome expression profiling, analysing the adaptive mechanisms initiated by *Mtb *in two common models of *Mtb *non-proliferation.
Betts *et al *(2002) [38] (Betts-expression)	Reports the use of gene and protein expression profiling to identify the response of *Mtb *to nutrient starvation, also modeling persistence.
Hampshire *et al *(2004) [41] (Hampshire-expression)	Reports the stationary phase gene expression of *Mtb *following a progressive nutrient depletion, proposing a model for persistence.

### Systems Analysis

#### Interactome Analysis

##### System Construction

We have constructed a protein-protein interaction network, based on the STRING database [17] version 7, which includes protein linkages between 3,925 *Mtb *proteins, inferred from published literature describing experimentally studied interactions, as well as those from genome analysis using several well-established methods such as domain fusion, phylogenetic profiling and gene neighbourhood concepts [18]. Thus, the network captures different types of interactions such as (a) physical complex formation between two proteins required to form a functional unit, (b) genes belonging to a single operon or to a common neighbourhood, (c) proteins in a given metabolic pathway and hence influenced by each other, (d) proteins whose associations are suggested based on predominant co-existence, co-expression, or domain fusion. Only the high-confidence interactions that had a STRING score of 0.7 or more were included in the network. We further augmented these with links between proteins that are influenced by the same metabolite, based on the reactions in the genome-scale metabolic reconstruction of *Mtb*, *iNJ*661 [19]. The resulting network contained 3,405 of the 3,925 proteins.

##### Node Deletions

Networks may be perturbed, through the removal of nodes and edges. A typical analysis would be to probe the effect of disrupting a node and its corresponding edges. Networks of different topologies vary in their resilience to various types of perturbations. The effect of node deletions on this network was analysed. Each of the 3,405 nodes was knocked out and the critical network parameters such as clustering coefficient and characteristic path length were monitored. In addition, the number of shortest paths that were disrupted in each deletion were monitored. The shortest paths between all pairs of proteins in the network were computed. Following removal of a node, some of these shortest paths may be disrupted, leading two pairs of nodes becoming unreachable from one another. Based on the change (loss) in the connectivity of nodes in this network and the change in network structure, on the deletion of nodes, we have delineated potential targets.

#### Reactome Analysis

Two independent genome-scale metabolic models for *Mtb *have become available. A genome-scale metabolic network, comprising 849 reactions, mediated by 739 metabolites and involving 726 genes, reported by McFadden and co-workers (GSMN-TB) [20] has been considered. Jamshidi and Palsson have reported another genome-scale metabolic model of *Mtb*, *iNJ*661, comprising 939 reactions mediated by 828 metabolites and 661 genes [19]. We have also earlier published a pathway-level model (MAP) of mycolic acid biosynthesis in *Mtb *[21]. We collated a list of lethal gene deletions for these studies. The essentiality predictions for the *iNJ*661 model were based on growth in Middlebrook 7H9 medium, as detailed in [19], while those for the GSMN-TB model were based on growth in Middlebrook 7H10 medium, as detailed in [20]. Genes whose deletion severely impaired growth (biomass formation) in the medium were designated as essential. The essentiality in [21] was studied using an objective function for optimal production of mycolates; a gene was considered essential if on deletion, most fluxes in the mycolic acid pathway including those of the mycolates dropped to zero. Using the COBRA Toolbox [22] for MATLAB, we also performed double gene deletions for the *iNJ*661 model.

#### Essentiality Analysis

Information on gene essentiality from a transposon site hybridisation (TraSH) mutagenesis study for *Mtb *[23] has also been incorporated in the decision criteria.

### Sequence Analysis

Close homologues for the *Mtb *proteins in the human proteome were identified by performing a BLAST search [24]. The BLAST results were parsed using python scripts based on BioPython . The criteria for regarding a protein as a close homologue were a sequence similarity of greater than 50% using a BLOSUM62 matrix, for a length of more than 50% of the bacterial query protein with an E-value less than 10^-4^.

### Structural Assessment of Targetability

#### Obtaining Structures

Crystal structures of 229 proteins from *Mtb *and 3,515 from human are available (excluding those with greater than 70% sequence identity) from the Protein Data Bank (PDB). This translates to a mere 6% of the *Mtb *proteome and under 10% of the human proteome. However, thousands of protein structures from both host and pathogen could be obtained using theoretically calculated structural models, from the ModBase database. Models in ModBase are built on the principles of homology modelling using Modeller [25]. Models of 2,808 proteins from *Mtb *and 16,000 proteins from the human proteome were obtained from this database. The database hosts multiple models for each protein, depending on the number of confident templates available for that protein in the PDB. For this analysis, only the first model for each protein was considered. Also, only those proteins which passed the previous stages of filtering in the target identification pipeline were considered. Of the 942 *Mtb *proteins considered, only 773 had available structures in ModBase.

#### Pocket Identification

In order to predict binding sites of a modelled protein, we have used PocketDepth (PD) [26], a geometry-based algorithm that has been developed and validated earlier by our group, to predict potential binding grooves on the surface of the protein. All possible binding sites in the 773 proteins of *Mtb *and the 16,000 human proteins were identified using PD. PD uses the concept of depth, which reflects how central a given pocket is and not merely how deep a subspace is in the pocket. PD outputs predicted binding sites in the form of sets or clusters. From such clusters, protein neighbourhoods within 4.0Å are extracted to obtain the binding sites.

An additional method to identify binding pockets in protein structures was used to obtain a consensus prediction. LigsiteCSC [27], a geometric method based on vectors in eight directions on a grid, also incorporating amino acid conservation information within each protein family, was used for this purpose. Top ten PD clusters were first obtained for each protein, which were compared with the top three pockets obtained from LigsiteCSC. Only the common clusters were retained for further analysis. 767 of the *Mtb *proteins and 15,830 of the human proteins were feasible for analysis, by which 3,500 pockets were identified in *Mtb *and 70,149 pockets in human.

#### Pocket Comparison

The next step towards structural assessment of targetability is to compare the binding sites of shortlisted targets of *Mtb *with those of the human proteome. An algorithm developed by us very recently, PocketMatch (PM) [28], has been used for this purpose. PM is based on shape signatures encoded by 90 lists of all-pair distances of residues in the binding site, pre-classified into one of the five standard amino acid types. A similarity score is assigned to each pair of binding sites. Extensive validation for PM, using the PDBbind database [29] of experimentally determined protein-ligand complexes is reported elsewhere [28]. We have now tested the algorithm to compare predicted pockets of all proteins in PDBbind as well. The SCOP-PM comparison for predicted pockets at various thresholds is provided as supplementary material [See Additional file 1].

All the 3,500 identified sites from the 767 short-listed proteins from *Mtb *were compared with the 70,149 identified sites from 15,830 human proteins. The topmost score for every protein pair is then chosen to capture the highest similarity an *Mtb *protein has in any of its pockets with any human protein. The scores are then compared to a pre-defined threshold as discussed in the results section to infer similarity. The exhaustive pairwise comparison of pockets is highly computationally intensive and was carried out on a massively parallel BlueGene (configuration: 4096 2-way shared memory processor nodes: 8192 IBM PowerPC 440×5 processors operating at 700 MHz, running Linux).

### Further Analysis of Short-listed Targets

The short-listed targets were subjected to further analysis, to retain only those proteins that are highly targetable.

#### Transcriptome Analysis/Gene Expression

One of the critical factors influencing the choice of a target would be its expression. Expression profiles related to persistence have been incorporated in [16]. Based on the expression of the genes, we have further filtered our list of targets. For this, we have used data from Small and co-workers [30], who have analysed the expression of genes in ten different strains of *Mtb*, *M. tuberculosis *H37Rv and *M. tuberculosis *H37Ra using cDNA microarrays. We also use data from Kaufmann and co-workers [31], who have performed a genome-wide expression analysis of *Mtb *from clinical lung samples using DNA arrays, and Barry and co-workers [32], who report an expression analysis of *Mtb *under a wide range of conditions. Lists of expressed genes have been reported in [30,31], while in [32], the *z*-scores have been reported for gene expression, in each of the experiments. A gene passed this filter if it was reported to be expressed, by either of [30,31], or in at least one of the studies (where an inhibitor of metabolism was not introduced) reported in [32].

#### Comparison with 'Anti-targets'

About seven proteins have been reported to form a set of 'anti-targets' [33], viz. the human ether-à-go-go-related gene (hERG), the pregnane X receptor (PXR), constitutive androstane receptor (CAR), P-glycoprotein (P-gp), as well as membrane receptors like the adrenergic *α*_1*a*_, the dopaminergic D2, the serotonergic 5 – *HT*_2*c *_and the muscarinic *M*_1_. Unintentional binding of drugs to these proteins causes adverse effects, leading to their labelling as anti-targets. The sequences of 306 proteins in the human proteome corresponding to these anti-targets were fetched from the NCBI sequence database. The accession numbers of these protein sequences are provided as supplementary material [see Additional file 2]. The short-listed targets were compared to these anti-targets by standard sequence analysis.

#### Similarity to Gut Flora Proteins

A number of organisms are known to inhabit the gut of a normal healthy individual [34]. Inadvertent inhibition of proteins of these organisms is likely to result in side effects. In order to study this possibility, the short-listed *Mtb *proteins were compared to the proteins of the gut flora (296,017 proteins from 95 organisms), again by sequence analysis. Some of these organisms are *Bacteroides intestinalis*, *Bifidobacterium bifidum*, *Bifidobacterium longum *and *Lactobacillus salivarius*. A full list of the 95 organisms is provided as supplementary material [see Additional file 3].

#### Involvement in Persistence

*Mtb *has an unusual capacity to persist in the host at many levels. In the cellular level, it resides in macrophages that typically function to eliminate pathogens and at the systemic level, it resists clearance by the adaptive immunity of the host. Its clearance by anti-bacterials is also very slow [35]. It may be possible to address the problem of persistence by targeting those genes that are implicated in persistence. For example, isocitrate lyase is a well-known persistence factor in mice, whose disruption attenuated bacterial persistence [36]. *pcaA*, a cyclopropane synthase involved in mycolic acid biosynthesis has also been shown to be a requirement for long-term mycobacterial persistence and virulence in mice models of tubercular infection [37]. Targets that passed all the previous filters were examined for expression during persistence based on several microarray expression data [32,38-41].

#### Phylogenetic Profiling

Phylogenetic profiling was carried out against 707 fully sequenced bacterial genomes. First, a BLAST was run against each of the 707 genomes, for *Mtb*. The BLAST output was then parsed using python scripts, based on BioPython, to obtain the E-value of the best hit, with a match of more than 50% of the query length, for each sequence in *Mtb*. The E-values thus obtained were converted to scores between 0 and 1, with 0 representing a strong match and 1 representing a weak match. The score was calculated as -1/log(*E*). Hits with *E *> *e*^-4 ^were all neglected and given a score of 1.0. This is identical to the scoring scheme of Protein Link EXplorer (PLEX) [42], which however currently considers only 89 genomes. For each protein in *Mtb*, profile strings comprising scores for the hits of the proteins were generated. Each profile string thus encodes the presence or absence of each of the *Mtb *proteins and where present, the extent of similarity as well. A subset of these results, for 228 pathogenic genomes, was analysed to examine the broad-spectrum nature of an identified target.

#### Involvement in Drug Resistance

Proteins involved in emergence of resistance to anti-tubercular drugs have been analysed and reported by us recently [43]. The list of about 25 proteins closely connected to different pathways of resistance were obtained and used for analysis here.

## Results

**Figure 1:**
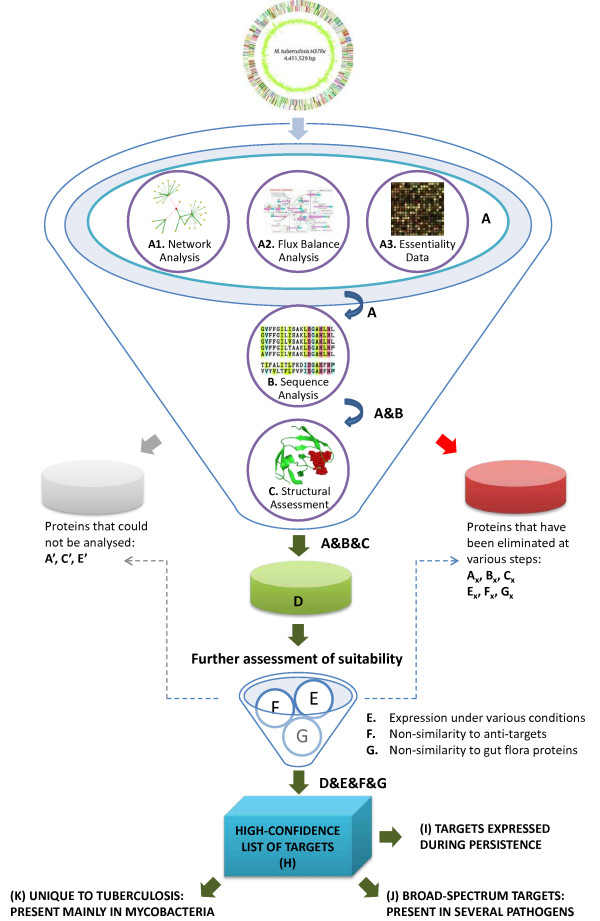
**The targetTB Target Identification Pipeline**. The funnel depicts the order in which the entire proteome of *Mtb *is considered and analysed at different layers. 'A' refers to the systems level studies, which includes A1, for network analysis of the interactome; A2, for flux balance analyses of the reactome; and A3, for genome-scale essentiality data determined experimentally as reported by Sassetti *et al *[23]. Those proteins that passed these filters are indicated as 'A', and combined with the results of sequence analysis (B), to derive those that passed both filters (depicted as 'A&B'). These were then taken through Filter C, referring to the structural assessment filter, yielding the list of 622 proteins as the D-List (A&B&C). Further steps of filtering are indicated in the smaller funnel as E (expression under various conditions), F (non-similarity to anti-targets) and G (non-similarity to gut flora proteins). Those proteins that pass all the six levels of filtering (indicated as D&E&F&G) form the H-List comprising 451 targets. Additional filters I, J and K used for analysing the H-List are also indicated. Lists A', C' and E' refer to the set of proteins at A, C and E levels, respectively, that could not be analysed for lack of appropriate data. Lists A_X_, B_X_, C_X_, E_X_, F_X_ and G_X_ refer to sets of proteins that failed in that particular filter, but may have passed at other levels.

**Figure 2:**
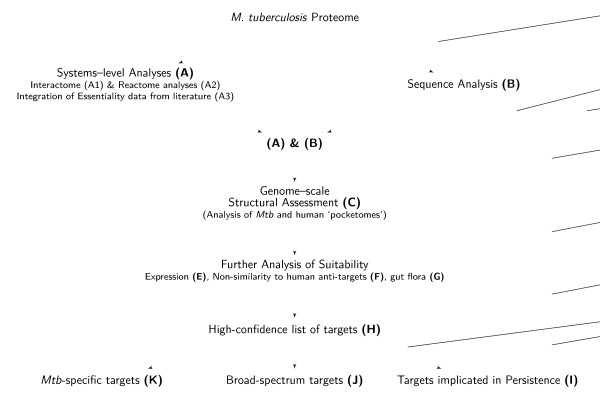
**Flowchart illustrating the sequence of analyses in this study**. This flowchart provides a simplified view of the various filters used in this study, in the order in which they are applied, to arrive at the final lists of targets.

A range of analyses spanning multiple levels of abstraction have been carried out, to identify plausible drug targets. The methodology can also be used more generally as a target identification pipeline that would be applicable to many drug discovery programmes. Starting from the entire proteome of *Mtb *H37Rv comprising 3,989 proteins, we have shortlisted 451 proteins as potential drug targets using a variety of filters, as depicted in Figs. 1 and 2. Fig. 1 illustrates a pictorial view of the targetTB pipeline while Fig. 2 shows a simplified view of the pipeline as a flowchart, illustrating the flow of this study. We first carry out a network analysis, where a full genome-scale interactome encoding several types of protein-protein interactions and protein-protein influences from metabolic pathways is reconstructed. Gene deletions that would significantly disrupt the network are then identified (List-A1). Next, we have studied the reactome through FBA (List-A2), to identify lethal gene deletions. This is further augmented with high-throughput gene essentiality data (List-A3). These system-level analyses together comprise Filter A. This is then integrated with sequence-level (Filter B) and structural analyses (Filter C) as described below (see Fig. 1). The expression of the gene encoding for the target is highly desirable (Filter E) and the list is further pruned by eliminating targets with high similarities to known 'anti-targets' in the human proteome (Filter F) and proteins in gut flora (Filter G). Those targets known to contribute to drug resistance in the pathogen are then prioritised. By analysis of similarity against several pathogenic proteomes, broad-spectrum targets as well as those unique to *Mtb *have also been identified. Various filters, lists and the numbers of proteins passed and eliminated at the various stages of the pipeline are given in Table 2.

**Table 2:** Models and methods used in the targetTB pipeline

**Analysis**	**Coverage**	**N/A**	✔	**X**		**Computation**
**(A) **Systems Analysis						
(i) Network	3405		431	2974		Single node deletions
(ii) FBA						
a) *Mtb iNJ*661	661		229	432		FBA
b) GSMN-TB	717		259	458		FBA
c) MAP	26		15	11		FBA
(iii) TraSH	3186		656	2530		
*Summary *((i) or (ii) or (iii))	3823	166 (A')	1138	2685	(A_X_)	

**(B) **Sequence Analysis	3989		3611	378	(B_X_)	3,989 *Mtb *vs. 33,453 human sequence comparisons

**(A&B) **Systems & Sequence Passed	3989	-	942	3047	(A_X_∪ B_X_)	

**(C) **Structural Assessment (PMScore < 0:8) [942 from A&B only considered]	767	175 (C')	622	145	(C_X_)	3,500 sites of *Mtb *(767 proteins) vs 70,149 sites of Human pocketome (15,830 proteins) = 245,521,500 pairwise comparisons

**(D) **A & B & C	3989	-	622	3552	(A_X_∪ B_X_∪ C_X_∪ C')	

**(E) **Expression						
(i) Gao-expression	3590	399	2210	1380		
(ii) Rachman-expression	634	3355	634	-		
(iii) Boshoff-expression	3915	74	3915	-		
*Summary *((i) or (ii) or (iii))	3917	72	3264	653	(E_X_)	
*Summary *(for (D))	622	1	529	92		

**(F) **Non-similarity to Anti-targets	3989	-	3928	61	(F_X_)	306 vs. 3,989 sequence comparisons
Non-similarity to Anti-targets (for (D))	622	-	611	11		

**(G) **Non-similarity to gut flora	3989	-	3730	259	(G_X_)	296,017 vs. 3,989 sequence comparisons
Non-similarity to gut flora (for (D))	622	-	543	79		

**(H) **D & E & F &; G	622	1	**451**	170		

**(I) **Expression during Persistence						
(i) Muttucumaru-expression	3924	82	639	3268		
(ii) Boshoff-expression	3915	74	105	3810		
(iii) Betts-expression	3649	340	274	3375		
(iv) Voskuil-expression	3924	82	310	3597		
(v) Hampshire-expression	3757	395	1349	2245		
*Summary *((i) or (ii) or (iii) or (iv) or (v))	3933	73	1871	2045		
*Summary *(for (H))	451	-	**216**	235		

**(J) **Broad-spectrum Targets [present in > 100/228 pathogenic genomes]	451	-	**186**	265		2,295,901 vs. 3,989 sequence comparisons (phylo-Genetic profiling against 707 genomes)

**(K) **Targets unique to Mycobacteria	451	-	**66**	385		

Number of proteins covered in each study are indicated. A '✔' indicates that it passes the filter, while a '**X**' indicates failure. The method used at each step is also indicated. The number of targets identified in the final lists H, I, J and K are boxed and indicated in bold typeface. The symbols A', C', A_X_-G_X_ are as described in the text and in Fig. 1.

### Systems Analysis

#### Interactome Analysis

A protein-protein interaction network comprising 3,405 nodes and 29,302 edges was constructed, which covered over 85% of the *Mtb *proteome. To evaluate the importance of a given protein in the context of the large interactome network, each node was individually deleted and its impact measured in terms of the number of shortest paths that are disrupted. Shortest paths in a network are quite critical to the structure of the network. Shortest paths in metabolic networks of *Mtb *and *M. leprae *have been identified and analysed by us earlier [44]. Samson and co-workers have earlier analysed a protein network in *Saccharomyces cerevisiae*, indicating that the analysis of shortest paths may provide an idea of network navigability as well as the efficiency with which a perturbation can spread throughout a network [45]. More recently, Wingender and co-workers have illustrated the importance of a similar metric, a 'pairwise disconnectivity index', for topological analysis of regulatory networks [46]. The disruption of shortest paths is expected to have a substantial effect on the network connectivity in protein networks as well. In the interactome network studied here, most of the node deletions do not significantly disrupt network connectivity. However, substantial effects (more than 5,000 disrupted shortest paths) were observed upon deletion of 431 of the 3,405 nodes (List-A1). These 431 proteins, for which a critical role in maintaining interactome network structure is suggested, were taken through further steps of filtering, in order to identify most useful drug targets. For example, for BirA (Rv3279c), close to 95,000 shortest paths in the network, were disrupted by its removal. A complete list of these proteins is provided as supplementary material [See Additional file 4].

#### Reactome Analysis

An FBA study, using the *iNJ*661 model [19], identified 188 proteins of the 661 studied, as essential for the growth of the bacterium, whereas an additional 41 also had a significant impact on growth (the *in silico *knock-out mutants were slow growers) [19]. A separate FBA study using an independently derived genome-scale metabolic model (GSMN-TB) identified 259 of the 719 proteins studied as essential for growth [20]. While these two models are similar in many respects, there are subtle differences in their biomass functions for FBA, as well as their coverage of the *Mtb *proteomes. 134 proteins were common to both lists of essential proteins. A third FBA study (MAP), carried out by us previously for the mycolic acid pathway alone identified 15 proteins in the pathway as essential for the microbe. Put together, the three studies suggest 318 proteins to be essential for the microbe. A critical role in maintaining the metabolism of the bacterium is suggested for the 318 proteins (List-A2). We have also carried out a double knockout study, on the *Mtb iNJ*661 model, identifying 49 pairs of genes, which when knocked out together, produce a lethal phenotype.

#### Essentiality Analysis

A high-throughput analysis of gene essentiality, using Transposon Site Hybridisation (TraSH) mutagenesis has been reported earlier. Genes, whose deletion produced slow-growing mutants, were also identified. These proteins (List-A3), taken together with Lists A1 and A2, form the list of proteins (List A) that are implicated to be essential, by systems-level analyses. We have combined the essentiality data, rather than take a consensus from the different system-level models discussed above, since each model has its own strengths and weaknesses. Many proteins are eliminated from the pipeline at this stage. For example, MabA (Rv1483), which has been suggested as a potential drug target [47,48], was not found to be essential in any of the systems-level analyses. MshA (Rv0486), suggested as an essential component of mycothiol biosynthesis and essential for growth in *Mtb *Erdman strain [49], is also not found to be essential in any of the systems-level studies.

### Sequence Analysis

At the sequence level, comparison with the human (host) proteome can be useful in filtering out those targets that have detectable homologues in the human cells, in order to reduce the risk of adverse effects that arise due to unintended interaction of the drug with the host protein. For 3,611 of 3,989 *Mtb *proteins, no close homologues were observed in the human proteome. The remaining 378 proteins, for which close homologues were observed, were eliminated at this step. The 3,611 proteins (List-B) were taken through further steps in the targetTB pipeline. Proteins such as KasA (Rv2245), KasB (Rv2246), MabA (Rv1483), RmlB (Rv3464), which have been suggested as potential targets in earlier studies, have all been eliminated at this stage, due to the presence of close homologues in the human proteome.

Combining the systems and sequence level analyses, 942 proteins were shortlisted for further analysis. A list of these proteins is presented as supplementary material [See Additional file 4].

### Structural Assessment of Targetability

Similarity between proteins is better captured through structural comparisons, where structural data for both proteins are available. In fact, what ultimately matters in determining the pharmacological profiles of drug molecules is the recognition of the drug molecules by various protein molecules at their binding sites. It is therefore important to compare binding sites in the various protein molecules in both the pathogen and the host. At this step, we want to critically weed out targets that share very high similarity with binding sites from the human 'pocketome', since targeting these may lead to adverse drug reactions, due to inadvertent binding with human proteins.

This type of analysis would become more meaningful if carried out at the proteome-scale. Advances in crystallography and various structural genomics projects [50-52] have led to the determination of 229 and 3,515 structures of *Mtb *and human, respectively. In the absence of experimentally determined structures, high-confidence homology models for 2,808 *Mtb *proteins and 16,000 human proteins were obtained from the ModBase database. The availability of such a large number of protein structures in both species makes it feasible to carry out a proteome-scale structural assessment of targetability. Identification of binding sites and further comparison of the identified binding sites are the next two challenging steps towards this goal. Two new algorithms that we have recently developed, PD and PM, enable us to carry out this comparison.

Of the 942 proteins shortlisted earlier in the pipeline, 773 had structures available in the PDB/ModBase databases. For these 773 proteins, the top 10 binding sites for each protein, identified using PD were compared with the top three binding pockets from LigsiteCSC. LigsiteCSC considers amino acid conservation at the putative sites, in the family of proteins. This automatically leads to identifying residues and hence the sites that are likely to be functionally important. Finding a consensus among top predictions between the two methods increases confidence in site prediction significantly. Some proteins such as DesA3 (Rv3229c), EmbB (Rv3795) and AccE5 (Rv3281) passed all other tests, but were not included in the H-List of high-confidence targets, since the structural analysis could not be performed.

A consensus between PD and LigsiteCSC was obtained so as to identify the most probable pockets that also contained conserved amino acid residues at the binding sites. Using this, 3,500 pockets were identified for 767 of the *Mtb *proteins. A similar exercise carried out for the human proteins identified 70,149 pockets. An all-versus-all comparison of the 'pocketomes' of *Mtb *and human was performed, using PM. This translated to 245,521,500 pairwise comparisons, which corresponded to over three years of serial CPU time, that was successfully completed on a BlueGene System, within a week.

A PM score of 0.8 or more indicates high similarity between two binding pockets. This threshold was used as a filter to eliminate all those proteins in *Mtb *whose pockets closely matched with any pocket of any protein in the human proteome. Of the 767 proteins, 145 had closely matching pockets in the human proteomes and were therefore eliminated from the pipeline. It is possible that some of these *Mtb *proteins contain some pockets that are sufficiently different from pockets of human proteins. Such proteins may also be targetable, but would require a close and more detailed analysis of all the pockets in the protein. The remaining 622 form a list of targets for anti-tubercular drugs. These proteins were taken through further steps of filtering to produce lists of highly viable targets.

Thus, of the 767 proteins that passed the A and B filters described above and had available structures, only 622 of them were found to pass this filter. This is despite the fact that sequence filtering was already carried out, re-emphasising the need for a multi-level target identification and validation scheme. The resulting proteins form the D-List, of targets that can be further explored for TB drug discovery.

### Further Analysis of Short-listed Targets

While the fundamental determinants of the quality of a target have already been considered earlier, the following aspects are also of importance in selecting a quality target for drug design. The following filters were therefore used to further prune the identified list and in some cases to enrich the list with targets having additional benefits.

#### Gene Expression

It is obvious that a target would be desirable only if it is expressed in the organism, at least under disease conditions. Expression data is available for over 3,900 genes in *Mtb *from various studies [30-32]. Of the shortlisted targets in the D-List, 529 are expressed, indicating their high viability as suitable targets. It must be noted here that the expression data are not comprehensive, especially in terms of the conditions that have been tested. The expression filter, while useful in understanding what is expressed and hence what is a useful target, should not be used to rule out otherwise useful targets. Until availability of more comprehensive data, this step is best used at the post-identification analysis stage. For example, proteins such as TrpD (Rv2192c), AroA (Rv3227), RibC (Rv1412) do not appear to be expressed in any of the experiments considered.

#### Comparison with Anti-targets

An ideal target should not only have specific recognition to the drug directed against it, but should also be sufficiently different from the host proteins, which have been termed as anti-targets. Considering this aspect early in the drug discovery pipeline may prove to be very useful in minimising the risk of failure of the drug candidates in the later stages of drug discovery. Anti-targets include proteins such as the transporters and pumps, which modify the bio-availability of a drug by their efflux action, or those proteins that trigger hazardous side effects, such as the hERG protein, which when blocked causes the 'sudden death syndrome' [33]. This list is by no means complete, but has been included here, more from a conceptual perspective, to highlight the need for screening against anti-targets. Sequence comparisons against 306 sequences belonging to the eight categories of anti-targets carried out revealed that sequence homologues at a similarity of 30% for over 30% of the query length were observed for 11 of the targets from the D-List. Such a loose similarity measure is used, since it is desired to rule out even a remote similarity with any anti-target. Moreover, close homologues have already been eliminated by sequence analysis earlier. A structural analysis of the proteins, when more data become available would be of immense utility in this regard. Serine/Threonine protein kinases such as the PknB (Rv0014c), earlier proposed as a target [53], PknL(Rv2176) and PknH (Rv1266c), as well as cytochromes such as Cyp128 (Rv2268c) and Cyp132 (Rv1394c) were eliminated at this stage.

#### Similarity to Gut Flora Proteins

The targets from the D-List were further compared to the protein sequences of hundreds of organisms that inhabit the gut of a healthy human. This was carried out to prune the list of identified drug targets, so that the drugs administered do not bind unintentionally to the proteins of the gut flora. Unintentional inhibition of gut flora proteins are known to lead to adverse effects and can promote pathogenic colonisation of the gut [54]. Drug interactions with gut flora are also believed to be the cause of idiosyncratic drug toxicity and reduced bio-availability of the drug [55,56]. Similarity of the identified targets to such proteins therefore affects their suitability. The sequence analyses carried out here indicate that 79 proteins from the D-List had close homologues in the gut flora and were hence removed from the list of most viable targets. For example, FtsZ (Rv2150c), Glf (Rv3809c) have homologues in gut flora and were hence eliminated at this stage. Interestingly, Icl (Rv0467), which has been particularly suggested as an attractive drug target [57] and also implicated in persistence [36], fails at this stage, due the presence of homologues in gut flora.

At this stage of filtering, from the 622 targets in the D-List identified earlier, 163 have been eliminated, leaving behind a high-confidence list of 451 targets (H-List). Several known targets appear in this list. A comprehensive analysis of the passage of several known targets in the targetTB pipeline has been performed. Some of these targets are indicated in Table 3, while the complete list is available as supplementary material [See Additional File 5].

**Table 3:** Results for known and proposed targets in the targetTB pipeline

**Target**	**Remarks**	**targetTB pipeline**									
		**A**	**B**	**C**	**E**	**F**	**G**	**H**	**I**	**J**	**K**
**I. Cell Wall Biosynthesis**											

DdlA (Rv2981c)	Known target of cycloserine [5]	✔	✔	**X**	✔	✔	✔	**X**			
EmbA (Rv3794)	Known target for ethambutol [7,75]	✔	✔	✔	✔	✔	✔	✔			
AftA (Rv3792)	Suggested as an attractive target [76]	✔	✔	**X**	✔	✔	✔	**X**			
AftB (Rv3805c)	Suggested as a potential target [77]	✔	✔	✔	✔	✔	✔	✔			
MurG (Rv2153c)	Suggested as a potential target [78]	✔	✔	✔	✔	✔	✔	✔	●	●	

**II. Lipid Metabolism**											

FabH (Rv0533c)	Possible target of thiolactomycin; also suggested as potential target [79,80]	✔	✔	✔	✔	✔	✔	✔		●	
FabD (Rv2243)	Suggested as a potential target [81-83]	✔	✔	✔	✔	✔	✔	✔		●	
AcpM (Rv2244)	Induced on isoniazid treatment [81,84]	✔	✔	✔	✔	✔	✔	✔	●		
Pks13 (Rv3800c)	Suggested as a promising target against *Corynebacterineae *[85]	✔	✔	✔	✔	✔	✔	✔	●		
InhA (Rv1484)	Known target for isoniazid, ethionamide [4]	✔	✔	✔	✔	✔	✔	✔		●	
PcaA (Rv0470c)	Suggested as a possible target of thiacetazone [86]	✔	✔	✔	✔	✔	✔	✔		●	
MmaA1 (Rv0645c)	-do-	✔	✔	✔	✔	✔	✔	✔	●	●	
FadD32 (Rv3801c)	Suggested as a promising target [87]	✔	✔	✔	✔	✔	✔	✔	●	●	
DesA3 (Rv3229c)	Suggested as a possible target [88]	✔	✔	**?**	✔	✔	✔	**X**			
Fas (Rv2524c)	Possible target of pyrazinamide [89]	✔	✔	✔	✔	✔	✔	✔			

**III. Intermediary Metabolism and Respiration**											

LysA (Rv1293)	Lysine auxotroph has vaccine potential [90]; suggested as potential target [91]	✔	✔	✔	✔	✔	✔	✔		●	
TrpD (Rv2192c)	-do-	✔	✔	✔	**X**	✔	✔	**X**			
LeuA (Rv3710)	Suggested as potential target [92]	✔	✔	✔	✔	✔	✔	✔	●		
DapB (Rv2773c)	Suggested as potential target [93]	✔	✔	✔	**X**	✔	**X**	**X**			
AroB (Rv2538c)	Shikimate pathway suggested as an attractive target [94]	✔	✔	✔	✔	✔	✔	✔	●	●	
ArgA (Rv2747)	Essential enzyme catalysing initial step of arginine biosynthesis [95]	✔	✔	✔	✔	✔	✔	✔			
AlrA (Rv3423c)	Known target of Cycloserine [5]	✔	✔	**X**	✔	✔	✔	**X**			
DfrA (Rv2763c)	Important drug target in many pathogens [96]. Suggested as drug target in [96,97]	✔	**X**	-	✔	✔	✔	**X**			
PanB (Rv2225)	Critical for pantothenic acid synthesis [98]	✔	✔	✔	✔	✔	✔	✔	●	●	
PanC (Rv3602c)	Critical for pantothenic acid synthesis [98]; suggested as potential target [99]	✔	✔	✔	✔	✔	✔	✔	●	●	
PanD (Rv3601c)	Critical for pantothenic acid synthesis [98]; suggested as potential target [100]	✔	✔	**X**	✔	✔	✔	**X**			
PanK (Rv1092c)/CoaA	Prokaryotic enzymes involved in the synthesis of CoA are good targets [101]; [102]	✔	✔	✔	✔	✔	✔	✔			
CysH (Rv2392)	Suggested as an attractive drug target [103-106]; CysH is important for *Mtb *protein during latent infection [58]	✔	✔	✔	✔	✔	✔	✔	●		
IspD (Rv3582c)	Potential drug target [107]	✔	✔	✔	✔	✔	✔	✔	●	●	
IspF (Rv3581c)	Potential drug target [107]; attractive target in many pathogens [59]	✔	✔	✔	✔	✔	✔	✔	●	●	
Icl (Rv0467)	Required for persistence of *Mtb *in macrophages and mice [36]; suggested as an attractive target [57]. Icl1 and Icl2 are required for fatty acid catabolism and virulence in *Mtb *[108]	✔	✔	✔	✔	✔	**X**	**X**			
AtpE1 (Rv1305)	Inhibited by a diarylquinoline drug R207910 *in vitro *[109]	✔	✔	✔	✔	✔	✔	✔			
Cyp121 (Rv2276)	Putative essential gene. Possible role in virulence through studies with ΔAraC/XylS gene regulator mutant (ΔRv1931c) [110]. Induced in isoniazid- and thiolactomycin-treated *Mtb *[111]	✔	✔	✔	✔	✔	✔	✔	●		

**IV. Information Pathways**											

GyrA (Rv0006)	Known target of uoroquinolones [112,113]	**X**	✔	-	✔	✔	**X**	**X**			
GyrB (Rv0005)	-do-	✔	✔	**X**	✔	✔	**X**	**X**			
RpoB (Rv0667)	Known target of rifampicin [8]	✔	✔	✔	✔	✔	**X**	**X**			
RpsL (Rv0683)	Known target of Streptomycin [114]	✔	**X**	-	✔	✔	**X**	**X**			

**V. Regulatory proteins**											

GlnE (Rv2221c)	Essential for growth of *Mtb *[115]	✔	✔	✔	✔	✔	✔	✔			
MtrA (Rv3246c)	Essential for growth of *Mtb *[116]	**X**	✔	-	✔	✔	✔	**X**			
DevR (Rv3133c)	Two-component system is a novel target in dormant mycobacteria [117]; essential for growth of *Mtb *under conditions of low oxygen [118]	✔	✔	✔	**X**	✔	✔	**X**			
DevS (Rv3132c)	Two-component system is a novel target in dormant mycobacteria [117]; part of the DevR-DevS two-component signal transduction system [118,119]	✔	✔	✔	✔	✔	✔	✔	●		●
PknB (Rv0014c)	Possibly essential for mycobacterial growth and hence possible target [53]	✔	✔	✔	✔	**X**	✔	**X**			
PknG (Rv0410c)	Crucial virulence factor [120]; possibly essential for mycobacterial growth and hence possible targets [53]	✔	✔	**X**	**X**	✔	✔	**X**			
MbtA (Rv2384)	An important adenylation enzyme required for siderophore biosynthesis [121]	✔	✔	**X**	✔	✔	✔	**X**			
IdeR (Rv2711)	Suggested as target [122,123]	**X**	✔	-	✔	✔	**X**	**X**			

**Figure 3:**
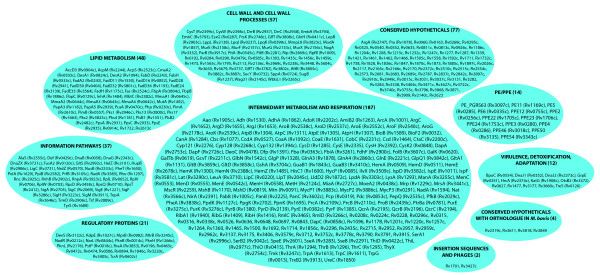
**List of Identified Targets**. Distribution of the functional classes of the 451 targets identified in the H-List. The number of targets present in each of the functional classes is also indicated.

An account of the passage of known targets (previously reported in literature) through the targetTB pipeline. The putative targets are classified based on their broad functional categories. A, B, C, E, F, G, H, I, J and K refer to the different filters depicted in Fig. 1 and described in the text. '✔' indicates that the given protein passes the filter, while a '**X**' indicates a failure. A '**?**' indicates that the analysis was not performed due to lack of appropriate data, while '-' indicates that the protein was not passed through the filter due to failure at a previous stage. All proteins in the H-List indicated in Fig. 3 would have a '✔' at levels A-H. '●' indicates the additional lists (I/J/K) in which a target from the H-List is present.

#### Involvement in Persistence

The expression of targets in the H-List, under conditions of persistence were analysed, from a set of microarray data. 216 of the H-List targets were up-regulated two-fold or more in at least one of the studies considered. These 216 targets form the I-List of targets, which may be useful in combating persistent *Mtb *infection. Some examples of proteins in the I-List are DesA1 (Rv0824c), DesA2 (Rv1094), DevS (Rv3132c), FadD32 (Rv3801c), KatG (Rv1908c), Pks13 (Rv3800c), CysH (Rv2392) and Wag31 (Rv2145c). CysH has also previously been shown to be important for *Mtb *persistence [58].

#### Identification of Broad-spectrum vs. Mtb-specific targets

Phylogenetic profiling of *Mtb *proteins against various genomes gives a measure of the uniqueness of a particular target to the *Mtb *proteome. Phylogenetic profiling can also help in identifying important functional linkages of chosen targets. It is also useful for identifying targets that can be used for designing broad-spectrum anti-bacterials. The 451 shortlisted targets were compared with 228 pathogenic bacterial genomes (provided as supplementary material [See Additional file 6]). If the *Mtb *target has close homologues in more than 100 genomes, we refer to it as a possible broad-spectrum anti-bacterial target (J-List). Several proteins involved in lipid metabolism are present in this list, viz. InhA (Rv1484), FabH (Rv0533c), FabD (Rv2243), PcaA (Rv0470c) and the MmaA's 1–4. IspF (Rv3581), which has been suggested as an attractive target in many pathogens [59], is also in the J-List. A main concern of such a strategy to target a multitude of bacteria in clinical therapy is the emergence of resistance to multiple organisms, which is highly undesirable. However, if the emergence of resistance is countered, as discussed below, having broad-spectrum targets could be of great advantage.

Proteins that were present only in mycobacteria were also identified by this analysis (K-List). This list is rich in mycobacteria PPE proteins and also contains proteins such as DevS, a sensor histidine kinase involved in a two-component signal transduction pathway.

#### Involvement in Drug Resistance

In a recent study, we identified possible pathways that would be involved in the emergence of drug resistance in *Mtb *[43]. We also proposed the concept of 'co-targets', referring to those proteins, which when inhibited simultaneously with a corresponding primary target, will help in reducing the emergence of resistance to the drug binding to that primary target. The importance of any protein in the H-List identified here will significantly increase if it also happens to be a constituent of the resistance pathways. These pathways comprise proteins that are predicted to be either directly responsible for generating resistance to the given drug, or serve as an important hub in the flow of information from the target of the given drug to the machinery of resistance. Proteins in the resistance pathways broadly belong to one of the four mechanisms, which are mediated by cytochromes, SOS related genes, antibiotic efflux pumps and genes involved in horizontal gene transfer (HGT). The putative targets in the H-List were analysed for their proximity to resistance-related proteins in the protein-protein interaction network described in [43]. Of 451 proteins in the H-List, 25 were closely involved in the resistance pathways and would therefore be significantly more useful as drug targets. Some notable examples are PolA (Rv1629), a protein involved in the SOS response, a cytochrome Cyp121 (Rv2276), which is also connected to 19 other cytochromes, and SecY (Rv0732), a protein connected to DnaE1 (SOS) and two other proteins, SecA1 and SecA2, implicated in HGT. Table 4 gives a list of these proteins and their association with resistance related proteins.

**Table 4:** Targets in the H-List that are also involved in drug resistance mechanisms.

**Resistance related proteins**	
**Protein**	**Description**
CcdA (Rv0527)	Cytochrome
PolA (Rv1629)	SOS
LldD2 (Rv1872c)	Cytochrome
QcrC (Rv2194)	Cytochrome
QcrB (Rv2196)	Cytochrome
CtaC (Rv2200c)	Cytochrome
Cyp121 (Rv2276)	Cytochrome
Rv3660c (Rv3660c)	HGT

**Proteins closely connected to resistance proteins**	

**Protein**	**Closely connected to**
SecY (Rv0732)	DnaE1 (SOS) and SecA1 and SecA2 (HGT)
Rv0843 (Rv0843)	Three cytochrome proteins
Pdc (Rv0853c)	Rv1988 (Antibiotic Efflux Pump) and two cytochromes
Rv1456c (Rv1456c)	Three cytochrome proteins
Rv1711 (Rv1711)	RecA, DnaE1 (SOS proteins) and a cytochrome
Rv1828 (Rv1828)	SecA2 (HGT) and two cytochromes
QcrC (Rv2194)	Four cytochrome proteins
QcrA (Rv2195)	Five cytochrome proteins
QcrB (Rv2196)	Five cytochrome proteins
CtaC (Rv2200c)	Five cytochrome proteins
Cyp121 (Rv2276)	19 cytochrome proteins
HemE (Rv2678c)	Seven cytochrome proteins
FtsK (Rv2748c)	SOS proteins RecA, PolA and DnaE1
RnhB (Rv2902c)	SOS proteins PolA, DnaE1 and DnaE2
TrmD (Rv2906c)	SOS proteins PolA, DnaE1 and Rv2294 (Antibiotic Efflux Pump)
PrfB (Rv3105c)	PolA (SOS) and SecA1 and SecA2 (HGT)
IlvX (Rv3509c)	Three cytochrome proteins
TrxB2 (Rv3913)	RecA (SOS) and two cytochrome proteins

The top panel shows targets that are directly implicated in resistance mechanisms. The lower panel indicates targets which are immediately connected to proteins involved in the emergence of resistance in the interactome. These proteins are predicted to be involved in mediating the flow of information from the targets to the resistance machinery.

### Targets Identified by the targetTB Pipeline

The various filters and the corresponding analyses that have been applied in this study, to arrive at the final lists of targets are listed in Table 2. Of the 3,989 proteins that have been annotated in the *Mtb *genome, 622 proteins pass the filters of systems and sequence analyses, as well as the structural assessment (D-List). These proteins are then screened to eliminate those which are not expressed, as well as those which have homologues in gut flora, or with anti-targets in the human proteome. A final list of 451 proteins is arrived at, which comprise the H-List. Of these, 216 proteins satisfy persistence criteria (I-List), while 186 are potential broad-spectrum anti-bacterial targets (J-List), and 66 targets are unique to mycobacteria (K-List). Proteins for which the analysis could not be performed, due to lack of available data at this time are separated as lists A' and C', which may be considered for analysis once more data become available. Proteins that have been eliminated at various stages could still find use as drug targets under different scenarios. For example, those proteins eliminated due to non-essentiality to *Mtb *(A_X_-List) may contain pairs of proteins that could together be essential and may hence be useful, if targeted concurrently. In fact, the double knock-out studies using FBA carried out here clear demonstrate this aspect. Similarly, proteins that have been eliminated due to some structural similarity with human targets (C_X_-List) may be useful as drug targets if the structural differences between the host and pathogen proteins could be exploited.

The functional classes of the 451 targets (H-List) identified by this study are indicated in Fig. 3. The list is also available as supplementary material [See Additional file 4]. This list includes several known targets and many that have been proposed as potential targets. Some known targets have been eliminated because they have failed one or more filters in the targetTB pipeline. The passage of known and proposed targets for anti-tubercular drugs in the targetTB pipeline is detailed in Table 3 (also see Additional File 5). Some examples of proteins that are in the H-List include known targets such as InhA, EmbA and FabH, as well as many targets that have been proposed for anti-tubercular drug discovery, such as GlfT2, a bi-functional UDP-galactofuranosyl transferase, the fatty acid synthase Fas, the pantothenate kinase PanK, a glutamine-synthetase adenylyltransferase GlnE and the sensor histidine kinase DevS. The list also indicates several proteins that have been suggested as potential drug targets in literature, but eliminated from the targetTB pipeline on account of failing one or more of the filters.

It is interesting to note that of the 451 targets in the H-List, over a half of them belong to the functional classes of 'lipid metabolism' and 'intermediary metabolism and respiration'. It has been said that metabolism has often not been given sufficient importance in 'intelligent' drug design [60]. Our analysis is in support of that observation, highlighting several targets from lipid metabolism, particularly the critical pathway of mycolic acid biosynthesis, amino acid biosynthesis, menaquinone biosynthesis and mycothiol biosynthesis. Several of the metabolites produced in these pathways are essential for mycobacterial survival and hence, the pathways producing these metabolites are ideal candidates for anti-tubercular drug discovery. Many of these pathways do not have equivalent pathways in the human, making them even more suitable candidates for targeting.

Desaturases DesA1 and DesA2, which have been shown by us to be hallmarks of the mycolic acid biosynthesis pathway in *Mtb *[61], pass all the filters and are present in the H-List. They are also present in the I-List of targets expressed during persistence. These proteins thus appear to be highly viable targets for anti-tubercular drugs. AcpS (Rv2523c), an acyl-carrier-protein synthase involved in mycolic acid biosynthesis, also passes all the filters and is a potential target. TrxB2 (Rv3913), a probable thioredoxin reductase and LysA (Rv1293), a diaminopimelate decarboxylase which catalyses the conversion of diaminopimelic acid to lysine and ThrB (Rv1296), a probable homoserine kinase, which are also ranked very high (ranked two, four and six, respectively) in the metabolic list of prioritised targets reported by Schreiber and co-workers [16], are also targets of interest.

### Comparison with Earlier Computational Studies

Two computational studies, outlining strategies for target identification, particularly for anti-tubercular drugs, have been reported earlier [15,16]. We present an overview of the passage of the targets suggested in these studies in the targetTB pipeline, also outlining the advantages of the targetTB pipeline over the previously reported methods.

#### Anishetty et al (2005) [15]

Based on a sequence analysis study, comparing enzymes in metabolic pathways between human and *Mtb*, Pennathur and co-workers proposed 186 proteins as suitable drug targets. Of these, 51 feature in our H-List and 129 do not, while six could not be considered for lack of sufficient functional data. Some examples of the 51 targets featuring in the H-List are AcpS (Rv2523c), AtpC (Rv1311), FabH (Rv0533c), FbpA (Rv3804c), FolB (Rv3607c), IspE (Rv1011), KatG (Rv1908c), LeuA (Rv3710), MenC (Rv0553), PanB (Rv2225), PanC (Rv3602c), PpdK (Rv1127c), GlfT1 (Rv3782) and TrpA (Rv1613). An account of how each of the 180 proteins proposed as targets in the study reported by Anishetty *et al *(2005) fare in the targetTB pipeline is given as supplementary material [See Additional file 7].

Of the 129 targets that do not pass the filters used in our study, but were predicted by Anishetty *et al*, 77 have been eliminated due to their non-essentiality in *Mtb*, as predicted by systems-level analyses, clearly demonstrating the need for incorporating systems-level studies. Of the remaining 52, one had a close homologue in the human proteome and 16 had a PM score of 0.8 or more, leading to their elimination. Of the remaining 35, 14 are not expressed under any of the conditions considered by the experiments considered (studies [30-32]), while 18 of them had homologues in gut flora (five failing both expression and gut flora filters). For the remaining eight, structural assessment through PD-LigsiteCSC-PM was infeasible due to lack of availability of an appropriate model. These observations reiterate the need for a comprehensive multi-level analysis for target identification, as demonstrated by the targetTB pipeline.

#### Hasan et al (2006) [16]

Schreiber and co-workers have reported a study in which they prioritise all proteins in the *Mtb *genome for use as drug targets. Their ranking is based on a consideration of metabolic choke-points, *in vitro *essentiality for growth and druggability as judged by sequence similarity to proteins capable of binding small molecule ligands, besides sequence analysis to identify unique proteins. Some concepts are similar between our study and that of Hasan *et al*, but our study differs from theirs in a number of ways: (i) to start with, the goal in our study is to identify a very high quality list of drug targets that are also computationally validated, whereas Hasan *et al *have aimed to prioritise all proteins in *Mtb *for their feasibility as drug targets (ii) a pipeline has been developed that filters out proteins at every stage, leading to a final list of very high quality targets at the same time eliminating the need for a blind consideration of all proteins at all stages. The pipeline is also useful for considering proteins eliminated at different steps, if required, with necessary caution. (iii) a rigorous FBA and network analysis have been carried out in our study, making the systems-level analysis much more comprehensive (iv) a comprehensive structural assessment of 767 proteins of *Mtb *that passed other filters in the pipeline, against 15,830 different human proteins, has been carried out. New algorithms developed by us have been used to identify and compare pockets, again rendering the structural analysis efficient and more importantly feasible, since it considers only the relevant features that describe drug recognition. In addition, we have considered (v) elimination of proteins similar to anti-targets and also (vi) those important in countering the emergence of drug resistance.

Hasan *et al *have proposed three lists of prioritised targets, based on different scoring schemes. In the metabolic list proposed by Hasan *et al*, 146 of the targets from the H-List are present in the top 500. Of the rest, 82 were eliminated due to the presence of sequence homologues in the human proteome. 107 were non-essential by systems analysis, while for eight, no data was available. Of the remaining 154, 43 were not feasible for structural analysis, while 49 had a PM Score of 0.8 or more. Two of the proteins had similarities with human anti-targets. Of the remaining 62, 36 had homologues in gut flora and 32 were not expressed (6 failed both filters). As a result, the final list of proteins that we have identified (H-List) differs significantly from those proposed by Hasan *et al*. A report of how the top 500 targets in each of the three lists proposed by Hasan *et al *(2006) fare in the targetTB pipeline is given as supplementary material [See Additional file 8].

## Discussion

It is now well-established that better insights into biological systems may be obtained by considering large-scale system-level models, since biological systems are complex networks of many processes. The conventional method of focussing on a single protein at a time, however important the protein may be, would mean losing perspective of its larger context and hence may not provide the right answers, especially in drug discovery. Broader insights about the appropriateness of a potential target can be obtained by considering pathways and whole-system models relevant to that disease. For example, an enzyme that may be identified as a good target for a particular disease may not actually be critical or essential, when viewed in the context of the entire metabolism in the cell. Analysing system-level models can help in assessing criticality of the individual proteins by studying any alternate pathways and mechanisms that may naturally exist to compensate for the absence of that protein. This study has demonstrated how systems biology can be used in drug target identification and drug discovery.

As the necessity of systems-level studies is becoming more and more obvious, a wide spectrum of techniques have been developed and applied for the simulation and analysis of biochemical systems [62-65]. These include stoichiometric techniques that rely on reaction stoichiometry and other constraints, kinetic pathway modelling techniques that rely on comprehensive mechanistic models and interaction-based analyses, as well as Petri nets and qualitative modelling formalisms [66]. The FBA carried out in conjunction with gene knock-outs here indicates the criticality of individual reactions and hence the associated proteins. In FBA, knock-outs can in fact be viewed as extreme inhibitions in which the target is totally inhibited by a drug. 188 of the 661 proteins in *Mtb iNJ*661 model resulted in lethal phenotypes when knocked out, indicating their essentiality for producing the required biomass and hence for bacterial growth. The FBA analysis also has the potential to consider multiple knock-outs again amounting to total inhibition at multiple points. Such a phenomenon is known to occur by some drugs individually and more commonly by a cocktail of drugs. For example, isoniazid is thought to act at two points in the pathway by inhibiting both InhA and KasA [4,67]. The FBA study presents a ready framework to analyse the effects of such drug inhibitions, which would be extremely difficult to judge by inspection of the reaction maps alone. Various combinations of the non-lethal gene deletions leading to about 111,628 different double knock-outs were generated and tested with FBA using the same objective function. 49 of them were found to lead to lethal phenotypes, with growth ratio of zero, as compared to that of the wild-type. Such proteins can be targeted simultaneously to achieve excellent antibacterial effect, although individually either one of them would not be good targets. Some examples of such pairs are Rv0505c (SerB1, non-essential)-Rv3042c (SerB2, in H-List), both phosphoserine phosphatases, Rv2243 (FabD, H-List)-Rv0649 (FabD2, non-essential), both malonyl CoA-ACP transacylases, Rv3273–Rv3588c, both carbonic anhydrases, and non-essential, individually, by systems analyses. It is conceivable that each of these pairs that appear to be isozymes produce a lethal phenotype on deletion, since the functional step of the pathways they catalyse may have proceeded in the absence of one, but would be arrested in the absence of both enzymes. Another example is that of Rv0363c (Fba, a fructose-bisphosphate aldolase)-Rv1237 (SugB, a sugar transport membrane protein ABC transporter). Such studies using FBA, however, can be carried out only for the annotated reactome component of the bacterial cell.

Networks obtained by considering various protein-protein interactions and influences, on the other hand are much more comprehensive and nearly complete in their coverage, especially because of the availability of an integrated database that considers experimentally mapped interactions and those predicted from one or more of the four well-established computational methods [17,18]. A drawback of such a network however, could be a large number of false positives. To minimise the introduction of false positives, we have eliminated all low-confidence interactions from our study. The number of broken paths introduced by a knock-out is taken here as a measure of the essentiality of the protein in maintaining the network. Biological networks typically display a power-law degree distribution. We explore the importance of the disruption of network connectivity that occurs on account of attacking nodes that lie on many shortest paths in the network. The advantage of interaction-based modelling such as this is that it is possible to generate interaction networks from existing databases and it is not constrained by lack of quantitative mechanistic data.

Besides essentiality to the pathogen, an ideal target should have several other properties such as non-similarity with human proteins whose inhibition could lead to potential adverse drug effects, an aspect that has been analysed at multiple levels in this study (see Fig. 1). The simplest level of course is to check for sequence similarity of the target being queried with all the proteins in the human proteome. Sequence information is readily available for hundreds of bacteria and this type of analysis is reported earlier for pathogenic genomes such as *Burkholderia pseudomallei *[68], *Helicobacteri pylori *[69], *Pseudomonas aeruginosa *[70,71] and even *Mtb *[72]. However, such sequence filtering while important, cannot be the sole criteria for identifying high quality targets, since two proteins that are considerably dissimilar in their sequences could have very similar binding sites [73,74]. Thus, while sequence similarity very often leads to structural and hence functional similarity, it is not a necessary condition for two proteins to have similar ligand binding profiles.

In the process of target identification, what really matters for a good target is to have a binding site in the target protein that is sufficiently different from that of any host protein. This is so that a given drug is both available in intended quantities to the intended target and perhaps more importantly, to avoid adverse effects by the drug binding to another protein from the host and manipulating its function as well, which is unintended and unanticipated. For this purpose, it is not very intuitive to look at structural classes and overall properties such as the structural family or secondary structural types, that might describe a structure. Instead, it is important to study the possible binding profile of a given drug to all those proteins to which it is likely to be exposed. Towards this goal, we first identified possible pockets in the set of *Mtb *and human structures, using PD, a validated algorithm that was recently developed in our lab. All such putative pockets were tested for certain criteria such as size and volume, retaining only those that were likely to bind to small molecules. The filtered pockets from preliminarily shortlisted targets from *Mtb *were then screened for similarity against pockets from the human proteins, which involved over 245 million comparisons, using PM, a site-matching algorithm recently developed in our laboratory. From this, 145 putative targets were eliminated due to high similarity with one or more human proteins. Interestingly, well-known molecules such as AlrA, PanD and GyrB are observed to have high similarities with proteins in the human, perhaps explaining the side effects caused by the drugs targeting them. With a cut-off in PMScore of 60%, molecules such as InhA, EmbA and EmbC, would all have been eliminated from the list for not having the properties of a safe target. However, since it is in principle, possible to design inhibitors that could bind only to the intended target by exploiting subtle structural differences that exist at the sites of the bacterial target in question with those of the human proteins obtained as hits with PM, we chose to use a high cut-off of 80%, so as to remove only those with very high risk of causing side effects. Some examples of molecules that have failed at this stage are DdlA, GyrB, AftA and AlrA. It must be noted that some of these were ranked as high priority targets by other studies that did not consider the structural aspect explicitly, again emphasising the need for structural level analysis. Eliminating those proteins with high similarity to proteins in the gut flora also helps in ultimately reducing the risk of side effects.

The last stages of filtering and post-identification analysis resulted in identifying two categories of targets: broad-spectrum targets and *Mtb*-specific targets. It is necessary to identify targets in both the categories, since they are required in different situations. *Mtb*-specific targets are believed to be safer since they would not lead to many organisms developing resistance against the drugs of such targets. Broad-spectrum targets, on the other hand, would be extremely useful when multiple infections co-exist or in some cases where a specific diagnosis is not possible. A comprehensive phylogenetic analysis of the shortlisted targets against 228 different pathogenic genomes has been carried out in this study, leading to the identification of broad-spectrum targets. Identification of pathways and proteins involved in generating drug resistance and then targeting them simultaneously as co-targets along with the primary broad-spectrum targets would reduce the risk of drug resistance significantly, making many more molecules accessible for therapeutic intervention.

## Conclusion

In summary, network analysis of the interactome in *Mtb *and flux balance analysis of the reactome, both systems-level studies, have helped in identifying a set of proteins critically required for the survival of the bacterium. By mapping these with experimentally determined essentiality data, a set of proteins that would be useful as drug targets is identified. The list is pruned by a series of filters to eliminate all those with a risk of causing side effects. Traditionally, drug safety has been addressed by modification of the drug molecule itself, but this paper reports how a careful choice of the target molecule can be made to achieve that goal, which could be used as a general strategy right in the beginning of the drug discovery process. To our knowledge, this is also the first study to carry out a comprehensive structural level analysis of identifying binding pockets and matching them so as to obtain a possible pharmacodynamic map of the administered drugs. In addition to the sequence and structure level filters, the final list of targets identified has also passed filters put in place to eliminate those similar to known anti-targets and the gut flora proteins. Finally, the list is further enriched by considering possible mechanisms in emergence of drug resistance. The pipeline developed provides rational schema for drug target identification that are likely to have high rates of success, which should save enormous amounts of money, resources and time in the drug discovery process.

## Abbreviations

FBA: Flux Balance Analysis; HGT: Horizontal Gene Transfer; *Mtb*: *Mycobacterium tuberculosis*; PD: PocketDepth; PM: PocketMatch; TB: tuberculosis.

## Authors' contributions

NC generated the idea and closely supervised the project. KR performed most of the work in this study, especially that of systems and sequence-level analyses. YK performed the structural comparisons and validations. KR and NC wrote the manuscript and all authors read and approved the final manuscript.

## Supplementary Material

Additional file 1 **Validation of PocketMatch for predicted pockets.** Figure illustrating the variation of the XOR of PocketMatch matrix vs. SCOP matrix, for various PM thresholds. Click here for file

Additional file 2 **Anti-target sequences.** Accession numbers of the 306 anti-target sequences considered. Click here for file

Additional file 3 **List of gut flora.** List of 95 organisms present in gut flora, as retrieved from the NCBI database. Click here for file

Additional file 4 **Lists of proteins passing various filters.** Detailed lists of proteins passing various filters. Also includes the final lists H, I, J and K. Click here for file

Additional file 5 **Passage of known and proposed targets in the targetTB pipeline.** An account of the passage of known targets (previously reported in literature) through the targetTB pipeline. The putative targets are classified based on their broad functional categories. Click here for file

Additional file 6 **List of pathogenic genomes.** List of 228 pathogenic genomes considered. Click here for file

Additional file 7 **Comparison with Anishetty *et al *(2005).** A report of how the proteins proposed as targets in the study reported by Anishetty *et al *(2005) fare in the targetTB pipeline. Click here for file

Additional file 8 **Comparison with Hasan *et al *(2005).** A report of how the top 500 targets in each of the three lists proposed by Hasan *et al *(2006) fare in the targetTB pipeline. Click here for file
