# Supplementary material for: targetTB: A target identification pipeline for Mycobacterium tuberculosis through an interactome, reactome and genome-scale structural analysis
Source: BMC Syst Biol. 2008 Dec 19;2:109. doi: 10.1186/1752-0509-2-109 (PMC2651862; doi:10.1186/1752-0509-2-109)
Supplement: Additional file 1 — Validation of PocketMatch for predicted pockets. Figure illustrating the variation of the XOR of PocketMatch matrix vs. SCOP matrix, for various PM thresholds. [file 1752-0509-2-109-S1.pdf]

SCOP-based Validation of PocketMatch for predicted pockets

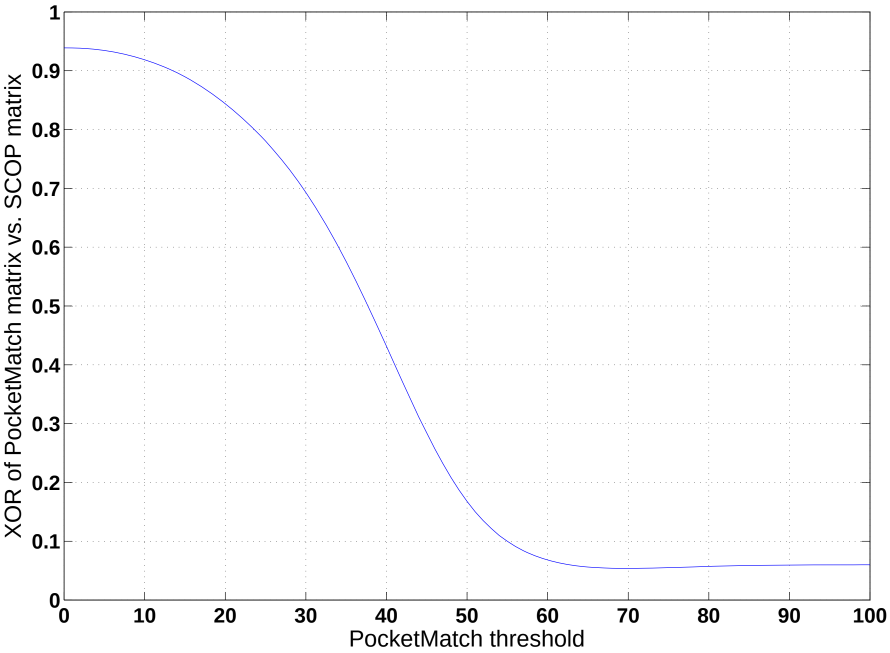

Figure 1: **Variation of XOR of PocketMatch matrix vs. SCOP matrix for various PocketMatch thresholds.** Plot showing the XOR values for different PMScore-SCOP matrix pairs for various PocketMatch (PM) thresholds, from 0 to 1, in steps of 0.01. The dataset used is PDBbind, comprising 954 proteins with 3,225 predicted clusters that overlap with ligands selected after pruning small ligands (<6 atoms), covalently attached ligands (within 2.0Å from any protein atom) and those that are marked for removal by Gold and Jackson [1]. The pockets for each of the 954 proteins were identified using PocketDepth [2]. An all-versus-all comparison of these pockets was then carried out and a PM score assigned to each of the protein pairs. Two proteins were considered as similar or dissimilar, based on a given threshold and given a score of 1 or 0, to construct an all-versus-all score matrix. A matrix of an identical size was constructed for the same set of proteins, based on their SCOP (Structural Classification of Proteins) [3] similarities, again assigning values of 1 or 0, indicating similar or dissimilar SCOP classes up to four levels, respectively. The two matrices were then directly compared and the number of matches were obtained. The same comparison was carried out for different thresholds of PM scores. It is clear from the figure that at thresholds of 0.65 and higher, the agreement with the SCOP matrix is also higher, reaching a value of about 95% agreement, at a PM threshold of about 0.70 and higher. PM algorithm is reported in [4].

References

[1] Gold ND, Jackson RM: **Fold independent structural comparisons of protein-ligand binding sites for exploring functional relationships**. *J Mol Biol* 2006, **355**(5):1112–1124.

[2] Kalidas Y, Chandra N: **PocketDepth: A new depth based algorithm for identification of ligand binding sites in proteins**. *J Struct Biol* 2008, **161**:31–42.

[3] Murzin AG, Brenner SE, Hubbard T, Chothia C: **SCOP: a structural classification of proteins database for the investigation of sequences and structures.** *J Mol Biol* 1995, **247**(4):536–540.

[4] Kalidas Y, Chandra N: **PocketMatch: A new algorithm to compare binding sites in protein structures**. *Available from Nature Precedings* 2008, : [http://hdl.handle.net/10101/npre.2008.2142.1].
