# Supplementary material for: targetTB: A target identification pipeline for Mycobacterium tuberculosis through an interactome, reactome and genome-scale structural analysis
Source: BMC Syst Biol. 2008 Dec 19;2:109. doi: 10.1186/1752-0509-2-109 (PMC2651862; doi:10.1186/1752-0509-2-109)
Supplement: Additional file 5 — Passage of known and proposed targets in the targetTB pipeline. An account of the passage of known targets (previously reported in literature) through the targetTB pipeline. The putative targets are classified based on their broad functional categories. [file 1752-0509-2-109-S5.pdf]

Passage of known and proposed targets in the targetTB pipeline

An account of the passage of known targets (previously reported in literature) through the targetTB pipeline. The putative targets are classified based on their broad functional categories. A, B, C, E, F, G, H, I, J and K refer to the different filters depicted in Fig. 1 and described in the text. '✓' indicates that the given protein passes the filter, while a '✗' indicates a failure. A '?' indicates that the analysis was not performed due to lack of appropriate data, while '–' indicates that the protein was not passed through the filter due to failure at a previous stage. All proteins in the H-list indicated in Fig. 3 would have a '✓' at levels A–H. '●' indicates the additional lists (I/J/K) in which a target from the H-List is present.

**Target**	**Remarks**	**targetTB pipeline**									
		A	B	C	E	F	G	H	I	J	K
**I. Cell Wall Biosynthesis**											
DdlA (Rv2981c)	Known target of cycloserine [1]	✓	✓	✗	✓	✓	✓	✗			
FtsZ (Rv2150c)	Suggested as possible drug target [2, 3]	✓	✓	✓	✓	✓	✗	✗			
Arabinogalactan biosynthesis											
EmbA (Rv3794)	Known target for ethambutol [4, 5]	✓	✓	✓	✓	✓	✓	✓			
EmbB (Rv3795)	–do–	✓	✓	?	✓	✓	✓	✗			
EmbC (Rv3793)	–do–	✓	✓	✓	✓	✓	✓	✓			
GlfT1 (Rv3782)	Suggested as possible target [6, 7]	✓	✓	✓	✓	✓	✓	✓			
GlfT2 (Rv3808c)	Suggested as important target for the development of new anti-tuberculosis drugs [8]; suggested as possible target [6, 7]	✓	✓	✓	✓	✓	✓	✓			
Glf (Rv3809c)	Suggested as important target for the development of new anti-tuberculosis drugs [8]	✓	✓	✓	✓	✓	✗	✗			
Peptidoglycan biosynthesis											
AftA (Rv3792)	Suggested as an attractive target [9]	✓	✓	✗	✓	✓	✓	✗			
AftB (Rv3805c)	Suggested as a potential target [10]	✓	✓	✓	✓	✓	✓	✓			
MurG (Rv2153c)	Suggested as a potential target [11]	✓	✓	✓	✓	✓	✓	✓	●	●	
MurX (Rv2156c)	–do–	✓	✓	✓	✓	✓	✓	✓	●	●	
RmlA (Rv0334)	Important in synthesis of dTDP-rhamnose [12]	✓	✓	✓	✗	✓	✗	✗			
RmlB (Rv3464)	Suggested as potential target [12]	✓	✗	–	✓	✓	✗	✗			
RmlC (Rv3465)	Suggested as potential target [12]	✓	✓	✓	✓	✓	✓	✓		●	
RmlD (Rv3266c)	Important in synthesis of dTDP-rhamnose [12]	✓	✓	✓	✓	✓	✓	✓	●	●	
Wag31 (Rv2145c)	Identified as novel target [13]	✓	✓	✓	✓	✓	✓	✓	●	●	
**II. Lipid Metabolism**											
Mycolic acid biosynthesis											
FabH (Rv0533c)	Possible target of thiolactomycin; also suggested as potential target [14, 15]	✓	✓	✓	✓	✓	✓	✓		●	
FabD (Rv2243)	Suggested as a potential target [16–18]	✓	✓	✓	✓	✓	✓	✓		●	
AcpM (Rv2244)	Induced on isoniazid treatment [16, 19]	✓	✓	✓	✓	✓	✓	✓	●		
Pks13 (Rv3800c)	Suggested as a promising target against *Corynebacterineae* [20]	✓	✓	✓	✓	✓	✓	✓	●		
InhA (Rv1484)	Known target for isoniazid, ethionamide [21]	✓	✓	✓	✓	✓	✓	✓		●	
MabA (Rv1483)	Recommended as a possible rational target [22, 23]	✗	✗	–	✓	✓	✓	✗			
KasA (Rv2245)	Possible target of thiolactomycin [24]/isoniazid [25]	✓	✗	–	✓	✓	✓	✗			
KasB (Rv2246)	Possible target of thiolactomycin [24]	✓	✗	–	✓	✓	✓	✗			
PcaA (Rv0470c)	Suggested as a possible target of thiacetazone [26]	✓	✓	✓	✓	✓	✓	✓		●	
CmaA2 (Rv0503c)	–do–	✓	✓	✓	✓	✓	✓	✓		●	
MmaA1 (Rv0645c)	–do–	✓	✓	✓	✓	✓	✓	✓	●	●	
MmaA2 (Rv0644c)	–do–	✓	✓	✓	✓	✓	✓	✓		●	
MmaA3 (Rv0643c)	–do–	✓	✓	✓	✓	✓	✓	✓		●	
MmaA4 (Rv0642c)	Suggested as a possible target of thiacetazone [26]; suggested as a possible target [27]	✓	✓	✓	✓	✓	✓	✓	●	●	
FadD32 (Rv3801c)	Suggested as a promising target [28]	✓	✓	✓	✓	✓	✓	✓	●	●	
AccD4 (Rv3799c)	Suggested as a promising target [28]	✓	✗	–	✓	✓	✓	✗			
AccA3 (Rv3285)	Suggested as a possible target [29]	✓	✗	–	✓	✓	✓	✗			
AccD5 (Rv3280)	Suggested as a possible target [29]	✓	✗	–	✓	✓	✗	✗			
AccE5 (Rv3281)	Suggested as a possible target [29]	✓	✓	?	✓	✓	✓	✗			
DesA3 (Rv3229c)	Suggested as a possible target [30]	✓	✓	?	✓	✓	✓	✗			
Fas (Rv2524c)	Possible target of pyrazinamide [31]	✓	✓	✓	✓	✓	✓	✓			
**III. Intermediary Metabolism and Respiration**											
AdoK (Rv2202c)	Suggested as a good bioactivator/pro-drug target [32]; suggested as potential drug target [33]	✓	✓	✓	✓	✓	✓	✓			
(Rv1347c)	Suggested as a valid drug target [34]	✓	✓	✓	✗	✓	✓	✗			
Dxr (Rv2870c)	Suggested as a highly promising drug target [35]	✓	✓	✗	✓	✓	✓	✗			
HadA (Rv0635)	HadAB is an exciting target [36]	✓	✓	✓	✓	✓	✓	✓	●		
HadB (Rv0636)	HadAB, HadBC are exciting targets [36]; likely target of dehydratase inhibitors in *M. bovis* [37, 38]	✓	✓	✓	✓	✓	✓	✓	●		
HadC (Rv0637)	HadBC is an exciting target [36]	✗	✓	–	✓	✓	✓	✗			

Target	Remarks	targetTB pipeline									
		A	B	C	E	F	G	H	I	J	K
HisE (Rv2122c)	Suggested as potential drug target [39]	✓	✓	✓	✗	✓	✗	✗			
Nat (Rv3566c)	Inhibition of Nat contributes to anti-mycobacterial activity of *Warburgia salutaris* [40]	✓	✓	✓	✓	✓	✓	✓			
ThyX (Rv2754c)	Suggested as potential target in several organisms [41]	✓	✓	✓	✓	✓	✓	✓			
Tmk (Rv3247c)	Suggested as potential drug target [42, 43]	✓	✓	✓	✓	✓	✓	✓	●		
Tpi (Rv1438)	Suggest the design of drug exploiting difference with the host enzyme [44]	✓	✗	–	✓	✓	✓	✗			
Nucleotide biosynthesis											
GuaB1 (Rv1843c)	Suggested as a drug target in several organisms [45]	✓	✓	✓	✓	✓	✓	✓	●	●	
GuaB2 (Rv3411c)	–do–	✓	✓	✗	✓	✓	✓	✗			
GuaB3 (Rv3410c)	–do–	✓	✓	✓	✓	✓	✓	✓	●		
RelA (Rv2583c)	Important in the survival of *Mtb* during nutrient starvation [46, 47]	✗	✓	–	✓	✓	✗	✗			
Amino acid biosynthesis											
Ald (Rv2780)	Suggested as potential drug target [48]	✓	✓	✗	✓	✓	✓	✗			
Asd (Rv3708c)	Suggested as an attractive drug target [49]	✓	✓	✓	✓	✓	✗	✗			
DapA (Rv2753c)	Stated as an important drug target [50]	✓	✓	✓	✓	✓	✓	✓		●	
DapC (Rv0858c)	Enzymes of lysine biosynthesis pathway are potential target candidates [51]	✓	✓	✓	✓	✓	✓	✓		●	
GlnA1 (Rv2220)	Essential for *Mtb* virulence [52]	✓	✓	✗	✓	✓	✗	✗			
LysA (Rv1293)	Lysine auxotroph has vaccine potential [53]; suggested as potential target [54]	✓	✓	✓	✓	✓	✓	✓		●	
LeuD (Rv2987c)	Δ*leuD* mutant unable to replicate in macrophages *in vitro* [55]	✓	✓	✓	✗	✓	✗	✗			
ProC (Rv0500)	Essential for *Mtb* virulence [56]	✓	✗	–	✓	✓	✓	✗			
TrpD (Rv2192c)	–do–	✓	✓	✓	✗	✓	✓	✗			
LeuA (Rv3710)	Suggested as potential target [57]	✓	✓	✓	✓	✓	✓	✓	●		
DapB (Rv2773c)	Suggested as potential target [58]	✓	✓	✓	✗	✓	✗	✗			
AroA (Rv3227)	Genes of the shikimate pathway suggested as potential targets [59–61]	✓	✓	✓	✗	✓	✓	✗			
AroB (Rv2538c)	Shikimate pathway suggested as an attractive target [62]	✓	✓	✓	✓	✓	✓	✓	●	●	
AroE (Rv2552c)	Suggested as a potential target [63]	✓	✓	✓	✓	✓	✓	✓	●		
AroF (Rv2540c)	Suggested as a potential target [64]	✓	✓	✓	✓	✓	✓	✓		●	
AroG (Rv2178c)	Genes of the shikimate pathway suggested as potential targets [59, 65]	✓	✓	✓	✓	✓	✓	✓			
AroK (Rv2539c)	Genes of the shikimate pathway suggested as potential targets [59, 60]	✓	✓	✓	✓	✓	✓	✓		●	
AroQ (Rv0948c)	Suggested as a promising target [66]	✗	✓	–	✓	✓	✓	✗			
*AroQ (Rv1885c)	Suggested as a novel target [67]; suggested as a promising target [66]	✓	✓	✓	✓	✓	✓	✓			
FbpB (Rv1886c)	Important promoter region for AroQ [66]	✓	✓	✓	✓	✓	✓	✓			
ArgA (Rv2747)	Essential enzyme catalysing initial step of arginine biosynthesis [68]	✓	✓	✓	✓	✓	✓	✓			
ArgC (Rv1652)	Suggested as potential target [69]	✓	✓	✓	✓	✓	✓	✓	●	●	
AlrA (Rv3423c)	Known target of Cycloserine [1]	✓	✓	✗	✓	✓	✓	✗			
Cofactor biosynthesis											
DfrA (Rv2763c)	Important drug target in many pathogens [70]. Suggested as drug target in [70, 71]	✓	✗	–	✓	✓	✓	✗			
PanB (Rv2225)	Critical for pantothenic acid synthesis [72]	✓	✓	✓	✓	✓	✓	✓	●	●	
PanC (Rv3602c)	Critical for pantothenic acid synthesis [72]; suggested as potential target [73]	✓	✓	✓	✓	✓	✓	✓	●	●	
PanD (Rv3601c)	Critical for pantothenic acid synthesis [72]; suggested as potential target [74]	✓	✓	✗	✓	✓	✓	✗			
PanK (Rv1092c) /CoaA	Prokaryotic enzymes involved in the synthesis of CoA are good targets [75]; [76]	✓	✓	✓	✓	✓	✓	✓			
Dfp (Rv1391) /CoaBC	Prokaryotic enzymes involved in the synthesis of CoA are good targets [75]	✓	✓	✓	✓	✓	✓	✓	●	●	
RibC (Rv1412)	Promising target [77, 78]; inhibition of enzymes involved in riboflavin biosynthesis provides a rational strategy for antibiotic drug design [79]	✓	✓	✓	✗	✓	✓	✗			
RibH (Rv1416)	–do–	✓	✓	✓	✓	✓	✓	✓	●	●	
Mycothiol biosynthesis											
MshA (Rv0486)	Essential for production of GlcNAc-Ins and growth in *Mtb* [80]; enzymes involved in mycothiol biosynthesis suggested as potential targets [81–84]	✗	✓	–	✓	✓	✓	✗			
MshB (Rv1170)	Important enzyme in mycothiol biosynthesis [85]; mycothiol biosynthetic pathway could constitute novel and important drug targets [83]; proposed as target [86]	✓	✓	✓	✓	✓	✓	✓			
MshC (Rv2130c)	Required for mycothiol production and is essential for *Mtb* survival [81]; Enzymes involved in mycothiol biosynthesis suggested as potential targets [81–84]	✓	✗	–	✓	✓	✓	✗			
MshD (Rv0819)	Enzymes involved in mycothiol biosynthesis suggested as potential targets [81–84]; survival of *Mtb* MshD mutants is severely compromised in activated and non-activated macrophages [87]	✓	✓	✓	✓	✓	✓	✓			
Sulphur Metabolism											
CysH (Rv2392)	Catalyses the first committed step in the biosynthesis of reduced sulphur compounds. CysH is actively expressed during the dormant phase of *Mtb* and in the environment of macrophages [88, 89]. Humans do not reduce sulfate for *de novo* cysteine biosynthesis and therefore do not have a CysH equivalent; hence can be an attractive drug target [90–93]; CysH is important for *Mtb* protein during latent infection [94]	✓	✓	✓	✓	✓	✓	✓	●		
Mca (Rv1082)	Critical role in mycobacterial detoxification of antibiotics [95]	✗	✓	–	✓	✓	✓	✗			
Sulphate assimilation enzymes											
CysT (Rv2399c)	Attractive targets, as many of these have no homologues in humans [90]	✓	✓	✓	✓	✓	✓	✓	●	●	
CysW (Rv2398c)	–do–	✓	✓	✓	✓	✓	✓	✓	●	●	
CysA (Rv2397c)	–do–	✓	✗	–	✓	✗	✗	✗			
SubI (Rv2400c)	–do–	✓	✓	✗	✓	✓	✓	✗			
CysN (Rv1286)	–do–	✓	✓	✗	✓	✓	✓	✗			
CysD (Rv1285)	–do–	✓	✓	✓	✓	✓	✓	✓	●		

Target	Remarks	A	B	C	E	F	G	H	I	J	K
CysC (Rv1286)	–do–	✓	✓	✗	✓	✓	✓	✗			
NirA (Rv2391)	–do–	✓	✓	✓	✓	✓	✓	✓	●		
CysK (Rv2334)	–do–	✗	✗	–	✓	✓	✗	✗			
CysM (Rv1336)	–do–	✗	✓	–	✓	✓	✓	✗			
CysM3 (Rv0848)	–do–	✓	✓	✓	✓	✓	✓	✓	●	●	
Terpenoid biosynthesis											
IspD (Rv3582c)	Potential drug target [96]	✓	✓	✓	✓	✓	✓	✓	●	●	
IspE (Rv1011)	–do–	✓	✓	✓	✓	✓	✓	✓		●	
IspF (Rv3581c)	Potential drug target [96]; attractive target in many pathogens [97]	✓	✓	✓	✓	✓	✓	✓	●	●	
Glyoxylate shunt											
Icl (Rv0467)	Required for persistence of *Mtb* in macrophages and mice [98]; suggested as an attractive target [99]. Icl1 and Icl2 are required for fatty acid catabolism and virulence in *Mtb* [100]	✓	✓	✓	✓	✓	✗	✗			
AceAB (Rv1916)		✗	✓	–	✓	✓	✓	✗			
CitE (Rv2498c)	May be useful as drug target [101]	✗	✗	–	✓	✓	✓	✗			
ATP Synthesis											
AtpE1 (Rv1305)	Inhibited by a diarylquinoline drug R207910 *in vitro* [102]	✓	✓	✓	✓	✓	✓	✓			
Menaquinone Biosynthesis											
MenA (Rv0534c)	Possibly an essential nutrient for *Mtb* [103]	✓	✓	?	✓	✓	✓	✗			
MenB (Rv0548c)	–do–	✓	✓	✓	✓	✓	✗	✗			
MenC (Rv0553)	–do–	✓	✓	✓	✓	✓	✓	✓			
MenD (Rv0555)	–do–	✓	✓	✓	✓	✓	✓	✓			
MenE (Rv0542c)	–do–	✓	✓	✓	✓	✓	✓	✓	●	●	
MenH (Rv0558)	–do–	✓	✓	✓	✓	✓	✓	✓		●	
Cytochrome P450s											
Cyp121 (Rv2276)	Putative essential gene. Possible role in virulence through studies with ΔAraC/XylS gene regulator mutant (ΔRv1931c) [104]. Induced in isoniazid- and thiolactomycin-treated *Mtb* [105]	✓	✓	✓	✓	✓	✓	✓	●		
Cyp125 (Rv3545c)	Induced in macrophages. Essential for infection in mice [106]	✗	✓	–	✓	✓	✓	✗			
Cyp128 (Rv2268c)	Possible essential gene. Required for *Mtb* growth *in vitro*. Expression upregulated post-starvation [104]	✓	✓	✓	✓	✗	✓	✗			
Cyp130 (Rv1256c)	Absent from *M. bovis* BCG vaccine strain (deletion RD13) [104]	✗	✓	–	✓	✓	✓	✗			
Cyp132 (Rv1394c)	Possible role in *Mtb* virulence. Transcription controlled by adjacent AraC transcriptional regulator [104, 107]	✓	✓	✓	✓	✗	✓	✗			
Cyp141 (Rv3121)	Absent from *M. bovis* BCG vaccine strain (deletion RD12) [104]	✗	✓	–	✓	✓	✓	✗			
Cyp144 (Rv1777)	Possible role in virulence [104]	✓	✓	✗	✗	✗	✓	✗			
**IV. Information Pathways**											
DNA Synthesis											
NrdB (Rv0233)	Ribonucleotide reductases (RNRs) are attractive targets for anti-proliferative drugs [108] and subunit vaccines [109] in other organisms [110]	✗	✓	–	✓	✓	✓	✗			
NrdE (Rv3051c)	*Mtb* RNR is a potential drug target; inhibition of RNR in a variety of mycobacterial species substantially alters the growth patterns of the organisms [111]; suggested as potential target [112] [110]	✓	✓	✗	✓	✓	✗	✗			
NrdF1 (Rv1981c)	[110]	✓	✓	✓	✓	✓	✗	✗			
NrdF2 (Rv3048c)	Potential target [112] [110]	✓	✓	✓	✓	✓	✗	✗			
LigA (Rv3014c)	Stated as a novel, validated and attractive drug target [113–115]; suggested as a possible drug target [116]	✓	✓	✓	✗	✓	✓	✗			
GyrA (Rv0006)	Known target of fluoroquinolones [117, 118]	✗	✓	–	✓	✓	✗	✗			
GyrB (Rv0005)	–do–	✓	✓	✗	✓	✓	✗	✗			
RpoB (Rv0667)	Known target of rifampicin [119]	✓	✓	✓	✓	✓	✗	✗			
RpsL (Rv0683)	Known target of Streptomycin [120]	✓	✗	–	✓	✓	✗	✗			
**V. Regulatory proteins**											
GlnE (Rv2221c)	Essential for growth of *Mtb* [121]	✓	✓	✓	✓	✓	✓	✓			
MtrA (Rv3246c)	Essential for growth of *Mtb* [122]	✗	✓	–	✓	✓	✓	✗			
DevR (Rv3133c)	Two-component system is a novel target in dormant mycobacteria [123]; essential for growth of *Mtb* under conditions of low oxygen [124]	✓	✓	✓	✗	✓	✓	✗			
DevS (Rv3132c)	Two-component system is a novel target in dormant mycobacteria [123]; part of the DevR-DevS two-component signal transduction system [124, 125]	✓	✓	✓	✓	✓	✓	✓	●		●
PknA (Rv0015c)	Possibly essential for mycobacterial growth and hence possible targets [126]	✓	✗	–	✓	✗	✓	✗			
PknB (Rv0014c)	–do–	✓	✓	✓	✓	✗	✓	✗			
PknG (Rv0410c)	Crucial virulence factor [127]; possibly essential for mycobacterial growth and hence possible targets [126]	✓	✓	✗	✗	✓	✓	✗			
PtpB (Rv0153c)	Possible target [128]	✗	✓	–	✗	✓	✓	✗			
Iron Acquisition											
MbtA (Rv2384)	An important adenylation enzyme required for siderophore biosynthesis [129]	✓	✓	✗	✓	✓	✓	✗			
IdeR (Rv2711)	Suggested as target [130, 131]	✗	✓	–	✓	✓	✗	✗			

References

[1] Feng Z, Barletta RG: **Roles of *Mycobacterium smegmatis* D-alanine:D-alanine ligase and D-alanine racemase in the mechanisms of action of and resistance to the peptidoglycan inhibitor D-cycloserine**. *Antimicrobial Agents and Chemotherapy* 2003, **47**:283–291.

[2] Respicio L, Nair PA, Huang Q, Anil B, Tracz S, Truglio JJ, Kisker C, Raleigh DP, Ojima I, Knudson DL, Tonge PJ, Slayden RA: **Characterizing septum inhibition in *Mycobacterium tuberculosis* for novel drug discovery.** *Tuberculosis* 2008, ***In Press***:doi:10.1016/j.tube.2008.03.001.

[3] Huang Q, Tonge PJ, Slayden RA, Kirikae T, Ojima I: **FtsZ: a novel target for tuberculosis drug discovery.** *Curr Top Med Chem* 2007, **7**(5):527–543.

[4] Belanger AE, Besra GS, Ford ME, Mikusova K, Belisle JT, Brennan PJ, Inamine JM: **The *embAB* genes of *Mycobacterium avium* encode an arabinosyl transferase involved in cell wall arabinan biosynthesis that is the target for the antimycobacterial drug ethambutol**. *Proceedings of the National Academy of Sciences* 1996, **93**(21):11919–11924.

[5] Escuyer VE, Lety MA, Torrelles JB, Khoo KH, Tang JB, Rithner CD, Frehel C, McNeil MR, Brennan PJ, Chatterjee D: **The Role of the embA and embB Gene Products in the Biosynthesis of the Terminal Hexaarabinofuranosyl Motif of *Mycobacterium smegmatis* Arabinogalactan**. *The Journal of Biological Chemistry* 2001, **276**(52):48854–48862.

[6] Rose NL, Zheng RB, Pearcey J, Zhou R, Completo GC, Lowary TL: **Development of a coupled spectrophotometric assay for GlfT2, a bifunctional mycobacterial galactofuranosyltransferase.** *Carbohydr Res* 2008, **343**(12):2130–2139.

[7] Beláňová M, Dianišková P, Brennan PJ, Completo GC, Rose NL, Lowary TL, Mikušová K: **Galactosyl transferases in mycobacterial cell wall synthesis.** *J Bacteriol* 2008, **190**(3):1141–1145.

[8] Pan F, Jackson M, Ma Y, McNeil MR: **Cell wall core galactofuran synthesis is essential for growth of mycobacteria**. *Journal of Bacteriology* 2001, **183**(13):3991–3998.

[9] Alderwick LJ, Seidel M, Sahm H, Besra GS, Eggeling L: **Identification of a novel arabinofuranosyltransferase (AftA) involved in cell wall arabinan biosynthesis in *Mycobacterium tuberculosis***. *The Journal of Biological Chemistry* 2006, **281**(23):15653–15661.

[10] Seidel M, Alderwick LJ, Birch HL, Sahm H, Eggeling L, Besra GS: **Identification of a novel arabinofuranosyltransferase AftB involved in a terminal step of cell wall arabinan biosynthesis in *Corynebacterianeae*, such as *Corynebacterium glutamicum* and *Mycobacterium tuberculosis***. *J Biol Chem* 2007, **282**(20):14729–14740.

[11] Hirano S, Ichikawa S, Matsuda A: **Design and synthesis of diketopiperazine and acyclic analogs related to the caprazamycins and liposidomycins as potential antibacterial agents.** *Bioorg Med Chem* 2008, **16**:428–436.

[12] Li W, Xin Y, McNeil MR, Ma Y: ***rmlB* and *rmlC* genes are essential for growth of mycobacteria**. *Biochemical and Biophysical Research Communications* 2006, **342**:170–178.

[13] Kang CM, Nyayapathy S, Lee JY, Suh JW, Husson RN: **Wag31, a homologue of the cell division protein DivIVA, regulates growth, morphology and polar cell wall synthesis in mycobacteria.** *Microbiology* 2008, **154**(Pt 3):725–735.

[14] Choi KH, Kremer L, Besra GS, Rock CO: **Identification and substrate specificity of β-ketoacyl (acyl carrier protein) synthase III (mtFabH) from *Mycobacterium tuberculosis***. *The Journal of Biological Chemistry* 2000, **275**:28201–28207.

[15] Musayev F, Sachdeva S, Scarsdale JN, Reynolds KA, Wright HT: **Crystal structure of a substrate complex of *Mycobacterium tuberculosis* β-ketoacyl-acyl carrier protein synthase III (FabH) with lauroyl-coenzyme A**. *Journal of Molecular Biology* 2005, **346**(5):1313–1321.

[16] Kremer L, Nampoothiri KM, Lesjean S, Dover LG, Graham S, Betts JC, Brennan PJ, Minnikin DE, Locht C, Besra GS: **Biochemical Characterization of Acyl Carrier Protein (AcpM) and Malonyl-CoA:AcpM Transacylase (mtFabD), Two Major Components of *Mycobacterium tuberculosis* Fatty Acid Synthase II**. *The Journal of Biological Chemistry* 2001, **276**:27967–27974.

[17] Zhang YM, White SW, Rock CO: **Inhibiting bacterial fatty acid synthesis**. *J Biol Chem* 2006, **281**(26):17541–17544.

[18] Ghadbane H, Brown AK, Kremer L, Besra GS, Fütterer K: **Structure of *Mycobacterium tuberculosis* mtFabD, a malonyl-CoA:acyl carrier protein transacylase (MCAT)**. *Acta Crystallogr Sect F Struct Biol Cryst Commun* 2007, **63**(Pt 10):831–835.

[19] Wilson M, DeRisi J, Kristensen HH, Imboden P, Rane S, Brown PO, Schoolnik GK: **Exploring drug-induced alterations in gene expression in Mycobacterium tuberculosis by microarray hybridization.** *Proc Natl Acad Sci U S A* 1999, **96**:12833–12838.

[20] Portevin D, Sousa-D'Auria CD, Houssin C, Grimaldi C, Chami M, Daffé M, Guilhot C: **A polyketide synthase catalyzes the last condensation step of mycolic acid biosynthesis in mycobacteria and related organisms**. *Proceedings of the National Academy of Sciences, USA* 2004, **101**:314–319.

[21] Banerjee A, Dubnau E, Quémard A, Balasubramanian V, Um KS, Wilson T, Collins D, de Lisle G, Jacobs Jr WR: ***inhA*, a gene encoding a target for isoniazid and ethionamide in *Mycobacterium tuberculosis*.** *Science* 1994, **263**:227–230.

[22] Cohen-Gonsaud M, Ducasse S, Hoh F, Zerbib D, Labesse G, Quémard A: **Crystal structure of MabA from *Mycobacterium tuberculosis*, a reductase involved in long-chain fatty acid biosynthesis**. *Journal of Molecular Biology* 2002, **320**(2):249–261.

[23] Marrakchi H, Ducasse S, Labesse G, Montrozier H, Margeat E, Emorine LJ, Charpentier X, Daffé M, Quémard A: **MabA (FabG1), a *Mycobacterium tuberculosis* protein involved in the long-chain fatty acid elongation system FAS-II**. *Microbiology* 2002, **148**:951–960.

[24] Kremer L, Douglas JD, Baulard AR, Morehouse C, Guy MR, Alland D, Dover LG, Lakey JH, Jacobs Jr WR, Brennan PJ,

Minnikin DE, Besra GS: **Thiolactomycin and related analogues as novel anti-mycobacterial agents targeting KasA and KasB condensing enzymes in *Mycobacterium tuberculosis***. *The Journal of Biological Chemistry* 2000, **275**(22):16857–16864.

[25] Mdluli K, Slayden RA, Zhu Y, Ramaswamy S, Pan X, Mead D, Crane DD, Musser JM, Barry III CE: **Inhibition of a *Mycobacterium tuberculosis* β-ketoacyl ACP Synthase by Isoniazid**. *Science* 1998, **280**:1607–1610.

[26] Alahari A, Trivelli X, Guérardel Y, Dover LG, Besra GS, Sacchettini JC, Reynolds RC, Coxon GD, Kremer L: **Thiacetazone, an antitubercular drug that inhibits cyclopropanation of cell wall mycolic acids in mycobacteria.** *PLoS ONE* 2007, **2**(12):e1343.

[27] Boissier F, Bardou F, Guillet V, Uttenweiler-Joseph S, Daffé M, Quémard A, Mourey L: **Further insight into S-adenosylmethionine-dependent methyltransferases: Structural characterization of Hma, an enzyme essential for the biosynthesis of oxygenated mycolic acids in *Mycobacterium tuberculosis***. *The Journal of Biological Chemistry* 2006, **281**(7):4434–4445.

[28] Portevin D, de Sousa-D'Auria C, Montrozier H, Houssin C, Stella A, Lanéelle MA, Bardou F, Guilhot C, Daffé M: **The Acyl-AMP Ligase FadD32 and AccD4-containing Acyl-CoA Carboxylase Are Required for the Synthesis of Mycolic Acids and Essential for Mycobacterial Growth**. *The Journal of Biological Chemistry* 2005, **280**(10):8862–8874.

[29] Gago G, Kurth D, Diacovich L, Tsai SC, Gramajo H: **Biochemical and structural characterization of an essential acyl coenzyme A carboxylase from *Mycobacterium tuberculosis***. *Journal of Bacteriology* 2006, **188**(2):477–486.

[30] Phetsuksiri B, Jackson M, Scherman H, McNeil MR, Besra GS, Baulard AR, Slayden RA, DeBarber AE, Barry III CE, Baird MS, Crick DC, Brennan PJ: **Unique Mechanism of Action of the Thiourea Drug Isoxyl on *Mycobacterium tuberculosis***. *The Journal of Biological Chemistry* 2003, **278**(52):53123–53130.

[31] Zimhony O, Cox JS, Welch JT, Vilchèze C, Jacobs Jr WR: **Pyrazinamide inhibits the eukaryotic-like fatty acid synthetase I (FASI) of *Mycobacterium tuberculosis*.** *Nat Med* 2000, **6**(9):1043–1047.

[32] Reddy MCM, Palaninathan SK, Shetty ND, Owen JL, Watson MD, Sacchettini JC: **High resolution crystal structures of *Mycobacterium tuberculosis* adenosine kinase: insights into the mechanism and specificity of this novel prokaryotic enzyme**. *J Biol Chem* 2007, **282**(37):27334–27342.

[33] Long MC, Shaddix SC, Moukha-Chafiq O, Maddry JA, Nagy L, Parker WB: **Structure–activity relationship for adenosine kinase from *Mycobacterium tuberculosis* II. Modifications to the ribofuranosyl moiety.** *Biochem Pharmacol* 2008, **75**(8):1588–1600.

[34] Frankel BA, Blanchard JS: **Mechanistic analysis of *Mycobacterium tuberculosis* Rv1347c, a lysine N^{ϵ}-acyltransferase involved in mycobactin biosynthesis.** *Arch Biochem Biophys* 2008, ***In Press***:doi: 10.1016/j.abb.2008.05.013.

[35] Henriksson LM, Unge T, Carlsson J, Åqvist J, Mowbray SL, Jones TA: **Structures of *Mycobacterium tuberculosis* 1-deoxy-D-xylulose-5-phosphate reductoisomerase provide new insights into catalysis**. *J Biol Chem* 2007, **282**(27):19905–19916.

[36] Sacco E, Covarrubias AS, O'Hare HM, Carroll P, Eynard N, Jones TA, Parish T, Daffé M, Bäckbro K, Quémard A: **The missing piece of the type II fatty acid synthase system from *Mycobacterium tuberculosis***. *Proc Natl Acad Sci U S A* 2007, **104**(37):14628–14633.

[37] Brown AK, Bhatt A, Singh A, Saparia E, Evans AF, Besra GS: **Identification of the dehydratase component of the mycobacterial mycolic acid-synthesizing fatty acid synthase-II complex.** *Microbiology* 2007, **153**(Pt 12):4166–4173.

[38] Brown AK, Papaemmanouil A, Bhowruth V, Bhatt A, Dover LG, Besra GS: **Flavonoid inhibitors as novel antimycobacterial agents targeting Rv0636, a putative dehydratase enzyme involved in *Mycobacterium tuberculosis* fatty acid synthase II.** *Microbiology* 2007, **153**(Pt 10):3314–3322.

[39] Javid-Majd F, Yang D, Ioerger TR, Sacchettini JC: **The 1.25 Å resolution structure of phosphoribosyl-ATP pyrophosphohydrolase from *Mycobacterium tuberculosis***. *Acta Crystallogr D Biol Crystallogr* 2008, **64**(Pt 6):627–635.

[40] Madikane VE, Bhakta S, Russell AJ, Campbell WE, Claridge TDW, Elisha BG, Davies SG, Smith P, Sim E: **Inhibition of mycobacterial arylamine N-acetyltransferase contributes to anti-mycobacterial activity of *Warburgia salutaris***. *Bioorg Med Chem* 2007, **15**(10):3579–3586.

[41] Chernyshev A, Fleischmann T, Kohen A: **Thymidyl biosynthesis enzymes as antibiotic targets**. *Appl Microbiol Biotechnol* 2007, **74**(2):282–289.

[42] Munier-Lehmann H, Chaffotte A, Pochet S, Labesse G: **Thymidylate kinase of *Mycobacterium tuberculosis*: a chimera sharing properties common to eukaryotic and bacterial enzymes.** *Protein Sci* 2001, **10**(6):1195–1205.

[43] van Daele I, Munier-Lehmann H, Froeyen M, Balzarini J, van Calenbergh S: **Rational Design of 5'-Thiourea-Substituted α-Thymidine Analogues as Thymidine Monophosphate Kinase Inhibitors Capable of Inhibiting Mycobacterial Growth**. *Journal of Medicinal Chemistry* 2007, **50**(22):5281–5292.

[44] Mathur D, Malik G, Garg LC: **Biochemical and functional characterization of triosephosphate isomerase from *Mycobacterium tuberculosis* H37Rv**. *FEMS Microbiology Letters* 2006, **263**(2):229–235.

[45] Shu Q, Nair V: **Inosine monophosphate dehydrogenase (IMPDH) as a target in drug discovery**. *Medicinal Research Reviews* 2008, **28**(2):219–232.

[46] Primm TP, Andersen SJ, Mizrahi V, Avarbock D, Rubin H, Barry III CE: **The stringent response of *Mycobacterium tuberculosis* is required for long-term survival**. *Journal of Bacteriology* 2000, **182**(17):4889–4898.

[47] Dahl JL, Kraus CN, Boshoff HI, Doan B, Foley K, Avarbock D, Kaplan G, Mizrahi V, Rubin H, Barry III CE: **The role**

of Rel$_{Mtb}$-mediated adaptation to stationary phase in long-term persistence of *Mycobacterium tuberculosis* in mice. *Proceedings of the National Academy of Sciences of the United States of America* 2003, **100**(17):10026–10031.

[48] Ågren D, Stehr M, Berthold CL, Kapoor S, Oehlmann W, Singh M, Schneider G: **Three-dimensional structures of apo- and holo-L-alanine dehydrogenase from *Mycobacterium tuberculosis* reveal conformational changes upon coenzyme binding.** *J Mol Biol* 2008, **377**(4):1161–1173.

[49] Singh A, Kushwaha HR, Sharma P: **Molecular modelling and comparative structural account of aspartyl β-semialdehyde dehydrogenase of *Mycobacterium tuberculosis* (H37Rv).** *J Mol Model* 2008, **14**(4):249–263.

[50] Mitsakos V, Dobson RCJ, Pearce FG, Devenish SR, Evans GL, Burgess BR, Perugini MA, Gerrard JA, Hutton CA: **Inhibiting dihydrodipicolinate synthase across species: towards specificity for pathogens?** *Bioorg Med Chem Lett* 2008, **18**(2):842–844.

[51] Weyand S, Kefala G, Weiss MS: **The three-dimensional structure of N-succinyldiaminopimelate aminotransferase from *Mycobacterium tuberculosis***. *J Mol Biol* 2007, **367**(3):825–838.

[52] Tullius MV, Harth G, Horwitz MA: **Glutamine synthetase GlnA1 is essential for growth of *Mycobacterium tuberculosis* in human THP-1 macrophages and guinea pigs.** *Infect Immun* 2003, **71**(7):3927–3936.

[53] Pavelka Jr MS, Chen B, Kelley CL, Collins FM, Jacobs Jr WR: **Vaccine efficacy of a lysine auxotroph of *Mycobacterium tuberculosis***. *Infection and Immunity* 2003, **71**(7):4190–4192.

[54] Gokulan K, Rupp B, Pavelka Jr MS, Jacobs Jr WR, Sacchettini JC: **Crystal structure of *Mycobacterium tuberculosis* diaminopimelate decarboxylase, an essential enzyme in bacterial lysine biosynthesis**. *The Journal of Biological Chemistry* 2003, **278**(20):18588–18596.

[55] Hondalus MK, Bardarov S, Russell R, Chan J, Jacobs Jr WR, Bloom BR: **Attenuation of and protection induced by a leucine auxotroph of *Mycobacterium tuberculosis***. *Infect Immun* 2000, **68**(5):2888–2898.

[56] Smith DA, Parish T, Stoker NG, Bancroft GJ: **Characterization of auxotrophic mutants of *Mycobacterium tuberculosis* and their potential as vaccine candidates**. *Infection and Immunity* 2001, **69**(2):1142–1150.

[57] Koon N, Squire CJ, Baker EN: **Crystal structure of LeuA from *Mycobacterium tuberculosis*, a key enzyme in leucine biosynthesis**. *Proceedings of the National Academy of Sciences of the United States of America* 2004, **101**(22):8295–8300.

[58] Paiva AM, Vanderwall DE, Blanchard JS, Kozarich JW, Williamson JM, Kelly TM: **Inhibitors of dihydrodipicolinate reductase, a key enzyme of the diaminopimelate pathway of *Mycobacterium tuberculosis***. *Biochimica et Biophysica Acta - Protein Structure and Molecular Enzymology* 2001, **1545**(1-2):67–77.

[59] Parish T, Stoker NG: **The common aromatic amino acid biosynthesis pathway is essential in *Mycobacterium tuberculosis***. *Microbiology* 2002, **148**(10):3069–3077.

[60] Oliveira JS, Pinto CA, Basso LA, Santos DS: **Cloning and overexpression in soluble form of functional shikimate kinase and 5-enolpyruvylshikimate 3-phosphate synthase enzymes from *Mycobacterium tuberculosis***. *Protein Expression and Purification* 2001, **22**(3):430–435.

[61] Pereira JH, De Oliveira JS, Canduri F, Dias MVB, Palma MS, Basso LA, Santos DS, De Azevedo Jr WF: **Structure of shikimate kinase from *Mycobacterium tuberculosis* reveals the binding of shikimic acid**. *Acta Crystallographica Section D: Biological Crystallography* 2004, **60**(12 II):2310–2319.

[62] de Mendonça JD, Ely F, Palma MS, Frazzon J, Basso LA, Santos DS: **Functional characterization by genetic complementation of aroB-encoded dehydroquinate synthase from *Mycobacterium tuberculosis* H37Rv and its heterologous expression and purification.** *J Bacteriol* 2007, **189**(17):6246–6252.

[63] Zhang X, Zhang S, Hao F, Lai X, Yu H, Huang Y, Wang H: **Expression, purification and properties of shikimate dehydrogenase from *Mycobacterium tuberculosis***. *Journal of Biochemistry and Molecular Biology* 2005, **38**(5):624–631.

[64] Ely F, Nunes JES, Schroeder EK, Frazzon J, Palma MS, Santos DS, Basso LA: **The *Mycobacterium tuberculosis* Rv2540c DNA sequence encodes a bifunctional chorismate synthase.** *BMC Biochem* 2008, **9**:13.

[65] Rizzi C, Frazzon J, Ely F, Weber PG, da Fonseca IO, Gallas M, Oliveira JS, Mendes MA, de Souza BM, Palma MS, Santos DS, Basso LA: **DAHP synthase from *Mycobacterium tuberculosis* H37Rv: cloning, expression, and purification of functional enzyme**. *Protein Expr Purif* 2005, **40**:23–30.

[66] Schneider CZ, Parish T, Basso LA, Santos DS: **The two chorismate mutases from both *Mycobacterium tuberculosis* and *Mycobacterium smegmatis*: biochemical analysis and limited regulation of promoter activity by aromatic amino acids.** *J Bacteriol* 2008, **190**:122–134.

[67] Agrawal H, Kumar A, Bal NC, Siddiqi MI, Arora A: **Ligand based virtual screening and biological evaluation of inhibitors of chorismate mutase (Rv1885c) from *Mycobacterium tuberculosis* H37Rv.** *Bioorg Med Chem Lett* 2007, **17**(11):3053–3058.

[68] Errey JC, Blanchard JS: **Functional characterization of a novel ArgA from *Mycobacterium tuberculosis***. *Journal of Bacteriology* 2005, **187**(9):3039–3044.

[69] Cherney LT, Cherney MM, Garen CR, Niu C, Moradian F, James MNG: **Crystal structure of N-acetyl-gamma-glutamyl-phosphate reductase from *Mycobacterium tuberculosis* in complex with NADP(+)**. *J Mol Biol* 2007, **367**(5):1357–1369.

[70] Gerum AB, Ulmer JE, Jacobus DP, Jensen NP, Sherman DR, Sibley CH: **Novel *Saccharomyces cerevisiae* screen identifies WR99210 analogues that inhibit *Mycobacterium tuberculosis* dihydrofolate reductase**. *Antimicrobial Agents and Chemotherapy* 2002, **46**(11):3362–3369.

[71] Li R, Sirawaraporn R, Chitnumsub P, Sirawaraporn W, Wooden J, Athappilly F, Turley S, Hol WGJ: **Three-dimensional**

structure of *M. tuberculosis* dihydrofolate reductase reveals opportunities for the design of novel tuberculosis drugs. *Journal of Molecular Biology* 2000, **295**(2):307–323.

[72] Sambandamurthy VK, Wang X, Chen B, Russell RG, Derrick S, Collins FM, Morris SL, Jacobs Jr WR: **A pantothenate auxotroph of *Mycobacterium tuberculosis* is highly attenuated and protects mice against tuberculosis**. *Nature Medicine* 2002, **8**(10):1171–1174.

[73] Velaparthi S, Brunsteiner M, Uddin R, Wan B, Franzblau SG, Petukhov PA: **5-tert-butyl-N-pyrazol-4-yl-4,5,6,7-tetrahydrobenzo[d]isoxazole-3-carboxamide derivatives as novel potent inhibitors of *Mycobacterium tuberculosis* pantothenate synthetase: initiating a quest for new antitubercular drugs.** *J Med Chem* 2008, **51**(7):1999–2002.

[74] Gopalan G, Chopra S, Ranganathan A, Swaminathan K: **Crystal structure of uncleaved L-aspartate-α-decarboxylase from *Mycobacterium tuberculosis***. *Proteins: Structure, Function, and Bioinformatics* 2006, **65**(4):796–802.

[75] Kumar P, Chhibber M, Surolia A: **How pantothenol intervenes in Coenzyme-A biosynthesis of *Mycobacterium tuberculosis*.** *Biochem Biophys Res Commun* 2007, **361**(4):903–909.

[76] Das S, Kumar P, Bhor V, Surolia A, Vijayan M: **Invariance and variability in bacterial PanK: A study based on the crystal structure of *Mycobacterium tuberculosis* PanK**. *Acta Crystallographica Section D: Biological Crystallography* 2006, **62**(6):628–638.

[77] Zhang Y, Jin G, Illarionov B, Bacher A, Fischer M, Cushman M: **A new series of 3-alkyl phosphate derivatives of 4,5,6,7-tetrahydro-1-D-ribityl-1H-pyrazolo[3,4-d]pyrimidinedione as inhibitors of lumazine synthase: design, synthesis, and evaluation.** *J Org Chem* 2007, **72**(19):7176–7184.

[78] Zhang Y, Illarionov B, Morgunova E, Jin G, Bacher A, Fischer M, Ladenstein R, Cushman M: **A new series of N-[2,4-dioxo-6-d-ribitylamino-1,2,3,4-tetrahydropyrimidin-5-yl]oxalamic acid derivatives as inhibitors of lumazine synthase and riboflavin synthase: design, synthesis, biochemical evaluation, crystallography, and mechanistic implications.** *J Org Chem* 2008, **73**(7):2715–2724.

[79] Talukdar A, Illarionov B, Bacher A, Fischer M, Cushman M: **Synthesis and enzyme inhibitory activity of the *S*-nucleoside analogue of the ribitylaminopyrimidine substrate of lumazine synthase and product of riboflavin synthase.** *J Org Chem* 2007, **72**(19):7167–7175.

[80] Buchmeier NA, Fahey RC: **The *mshA* gene encoding the glycosyltransferase of mycothiol biosynthesis is essential in *Mycobacterium tuberculosis* Erdman.** *FEMS Microbiol Lett* 2006, **264**:74–79.

[81] Sareen D, Newton GL, Fahey RC, Buchmeier NA: **Mycothiol Is Essential for Growth of *Mycobacterium tuberculosis* Erdman**. *Journal of Bacteriology* 2003, **185**(22):6736–6740.

[82] Vetting MW, Roderick SL, Yu M, Blanchard JS: **Crystal structure of mycothiol synthase (Rv0819) from *Mycobacterium tuberculosis* shows structural homology to the GNAT family of N-acetyltransferases**. *Protein Science* 2003, **12**(9):1954–1959.

[83] McCarthy AA, Peterson NA, Knijff R, Baker EN: **Crystal Structure of MshB from *Mycobacterium tuberculosis*, a Deacetylase Involved in Mycothiol Biosynthesis**. *Journal of Molecular Biology* 2004, **335**(4):1131–1141.

[84] Slättegård R, Gammon DW, Oscarson S: **Synthesis of fused bicyclic thioglycosides of N-acylated glucosamine as analogues of mycothiol.** *Carbohydr Res* 2007, **342**(12-13):1943–1946.

[85] Newton GL, Av-Gay Y, Fahey RC: **N-Acetyl-1-D-myo-inosityl-2-amino-2-deoxy-alpha-D-glucopyranoside deacetylase (MshB) is a key enzyme in mycothiol biosynthesis.** *J Bacteriol* 2000, **182**(24):6958–6963.

[86] Metaferia BB, Fetterolf BJ, Shazad-Ul-Hussan S, Moravec M, Smith JA, Ray S, Gutierrez-Lugo MT, Bewley CA: **Synthesis of natural product-inspired inhibitors of *Mycobacterium tuberculosis* mycothiol-associated enzymes: the first inhibitors of GlcNAc-Ins deacetylase**. *J Med Chem* 2007, **50**(25):6326–6336.

[87] Rengarajan J, Bloom BR, Rubin EJ: **Genome-wide requirements for *Mycobacterium tuberculosis* adaptation and survival in macrophages.** *Proc Natl Acad Sci U S A* 2005, **102**(23):8327–8332.

[88] Hampshire T, Soneji S, Bacon J, James BW, Hinds J, Laing K, Stabler RA, Marsh PD, Butcher PD: **Stationary phase gene expression of *Mycobacterium tuberculosis* following a progressive nutrient depletion: a model for persistent organisms?** *Tuberculosis* 2004, **84**(3-4):228–238.

[89] Schnappinger D, Ehrt S, Voskuil MI, Liu Y, Mangan JA, Monahan IM, Dolganov G, Efron B, Butcher PD, Nathan C, Schoolnik GK: **Transcriptional Adaptation of Mycobacterium tuberculosis within Macrophages: Insights into the Phagosomal Environment.** *J Exp Med* 2003, **198**(5):693–704.

[90] Schelle MW, Bertozzi CR: **Sulfate metabolism in mycobacteria**. *Chembiochem* 2006, **7**(10):1516–1524.

[91] Carroll KS, Gao H, Chen H, Stout CD, Leary JA, Bertozzi CR: **A conserved mechanism for sulfonucleotide reduction**. *PLoS Biology* 2005, **3**(8):–.

[92] Mougous JD, Senaratne RH, Petzold CJ, Jain M, Lee DH, Schelle MW, Leavell MD, Cox JS, Leary JA, Riley LW, Bertozzi CR: **A sulfated metabolite produced by stf3 negatively regulates the virulence of Mycobacterium tuberculosis.** *Proc Natl Acad Sci U S A* 2006, **103**(11):4258–4263.

[93] Williams SJ, Senaratne RH, Mougous JD, Riley LW, Bertozzi CR: **5'-adenosinephosphosulfate lies at a metabolic branch point in mycobacteria**. *The Journal of Biological Chemistry* 2002, **277**(36):32606–32615.

[94] Senaratne RH, Silva ADD, Williams SJ, Mougous JD, Reader JR, Zhang T, Chan S, Sidders B, Lee DH, Chan J, Bertozzi CR, Riley LW: **5'-Adenosinephosphosulphate reductase (CysH) protects Mycobacterium tuberculosis against free radicals during chronic infection phase in mice.** *Mol Microbiol* 2006, **59**(6):1744–1753.

[95] Newton GL, Av-Gay Y, Fahey RC: **A novel mycothiol-dependent detoxification pathway in mycobacteria**

involving mycothiol S-conjugate amidase. *Biochemistry* 2000, **39**(35):10739–10746.

[96] Eisenreich W, Bacher A, Arigoni D, Rohdich F: **Biosynthesis of isoprenoids via the non-mevalonate pathway**. *Cellular and Molecular Life Sciences* 2004, **61**(12):1401–1426.

[97] Buetow L, Brown AC, Parish T, Hunter WN: **The structure of Mycobacteria 2C-methyl-D-erythritol-2,4-cyclodiphosphate synthase, an essential enzyme, provides a platform for drug discovery**. *BMC Struct Biol* 2007, **7**:68.

[98] McKinney JD, zu Bentrup KH, Muñoz-Elías EJ, Miczak A, Chen B, Chan WT, Swenson D, Sacchettini JC, Jacobs Jr WR, Russell DG: **Persistence of *Mycobacterium tuberculosis* in macrophages and mice requires the glyoxylate shunt enzyme isocitrate lyase**. *Nature* 2000, **406**:735–738.

[99] Wang Y, Xiao CL: **Isocitrate lyase, a new target in anti-tuberculosis drug research**. *Chinese Journal of Antibiotics* 2007, **32**(7):391–395.

[100] Muñoz-Elías EJ, McKinney JD: ***Mycobacterium tuberculosis* isocitrate lyases 1 and 2 are jointly required for *in vivo* growth and virulence**. *Nature Medicine* 2005, **11**(6):638–644.

[101] Goulding CW, Bowers PM, Segelke B, Lekin T, Kim CY, Terwilliger TC, Eisenberg D: **The structure and computational analysis of *Mycobacterium tuberculosis* protein CitE suggest a novel enzymatic function**. *J Mol Biol* 2007, **365**(2):275–283.

[102] Andries K, Verhasselt P, Guillemont J, Gohlmann HWH, Neefs JM, Winkler H, Van Gestel J, Timmerman P, Zhu M, Lee E, Williams P, de Chaffoy D, Huitric E, Hoffner S, Cambau E, Truffot-Pernot C, Lounis N, Jarlier V: **A Diarylquinoline Drug Active on the ATP Synthase of *Mycobacterium tuberculosis***. *Science* 2005, **307**(5707):223–227.

[103] Mdluli K, Spigelman M: **Novel targets for tuberculosis drug discovery.** *Curr Opin Pharmacol* 2006, **6**(5):459–467.

[104] McLean KJ, Dunford AJ, Neeli R, Driscoll MD, Munro AW: **Structure, function and drug targeting in *Mycobacterium tuberculosis* cytochrome P450 systems.** *Arch Biochem Biophys* 2007, **464**(2):228–240.

[105] Betts JC, McLaren A, Lennon MG, Kelly FM, Lukey PT, Blakemore SJ, Duncan K: **Signature gene expression profiles discriminate between isoniazid-, thiolactomycin-, and triclosan-treated *Mycobacterium tuberculosis***. *Antimicrob Agents Chemother* 2003, **47**(9):2903–2913.

[106] Kendall SL, Rison SCG, Movahedzadeh F, Frita R, Stoker NG: **What do microarrays really tell us about *M. tuberculosis*?** *Trends Microbiol* 2004, **12**(12):537–544.

[107] Recchi C, Sclavi B, Rauzier J, Gicquel B, Reyrat JM: ***Mycobacterium tuberculosis* Rv1395 is a class III transcriptional regulator of the AraC family involved in cytochrome P450 regulation.** *J Biol Chem* 2003, **278**(36):33763–33773.

[108] Szekeres T, Fritzer-Szekeres M, Elford HL: **The enzyme ribonucleotide reductase: target for antitumor and anti-HIV therapy.** *Crit Rev Clin Lab Sci* 1997, **34**(6):503–528.

[109] Fagan PK, Walker MJ, Chin J, Eamens GJ, Djordjevic SP: **Oral immunization of swine with attenuated *Salmonella typhimurium aroA* SL3261 expressing a recombinant antigen of *Mycoplasma hyopneumoniae* (NrdF) primes the immune system for a NrdF specific secretory IgA response in the lungs.** *Microb Pathog* 2001, **30**(2):101–110.

[110] Dawes SS, Warner DF, Tsenova L, Timm J, McKinney JD, Kaplan G, Rubin H, Mizrahi V: **Ribonucleotide Reduction in *Mycobacterium tuberculosis*: Function and Expression of Genes Encoding Class Ib and Class II Ribonucleotide Reductases**. *Infection and Immunity* 2003, **71**(11):6124–6131.

[111] Yang F, Lu G, Rubin H: **Isolation of ribonucleotide reductase from *Mycobacterium tuberculosis* and cloning, expression, and purification of the large subunit**. *Journal of Bacteriology* 1994, **176**(21):6738–6743.

[112] Nurbo J, Roos AK, Muthas D, Wahlström E, Ericsson DJ, Lundstedt T, Unge T, Karlén A: **Design, synthesis and evaluation of peptide inhibitors of *Mycobacterium tuberculosis* ribonucleotide reductase**. *Journal of Peptide Science* 2007, **13**(12):822–832.

[113] Srivastava SK, Dube D, Kukshal V, Jha AK, Hajela K, Ramachandran R: **NAD+-dependent DNA ligase (Rv3014c) from *Mycobacterium tuberculosis*: novel structure-function relationship and identification of a specific inhibitor.** *Proteins* 2007, **69**:97–111.

[114] Srivastava SK, Tripathi RP, Ramachandran R: **NAD+-dependent DNA ligase (Rv3014c) from *Mycobacterium tuberculosis*: Crystal structure of the adenylation domain and identification of novel inhibitors**. *The Journal of Biological Chemistry* 2005, **280**(34):30273–30281.

[115] Srivastava SK, Dube D, Tewari N, Dwivedi N, Tripathi RP, Ramachandran R: ***Mycobacterium tuberculosis* NAD+-dependent DNA ligase is selectively inhibited by glycosylamines compared with human DNA ligase I**. *Nucleic Acids Research* 2005, **33**(22):7090–7101.

[116] Gong C, Martins A, Bongiorno P, Glickman MS, Shuman S: **Biochemical and genetic analysis of the four DNA ligases of mycobacteria**. *The Journal of Biological Chemistry* 2004, **279**(20):20594–20606.

[117] Aubry A, Pan XS, Fisher LM, Jarlier V, Cambau E: ***Mycobacterium tuberculosis* DNA Gyrase: Interaction with Quinolones and Correlation with Antimycobacterial Drug Activity**. *Antimicrob. Agents Chemother.* 2004, **48**(4):1281–1288.

[118] Mdluli K, Ma Z: ***Mycobacterium tuberculosis* DNA gyrase as a target for drug discovery.** *Infect Disord Drug Targets* 2007, **7**(2):159–168.

[119] Telenti A, Imboden P, Marchesi F, Matter L, Schopfer K, Bodmer T, Lowrie D, Colston MJ, Cole ST: **Detection of rifampicin-resistance mutations in *Mycobacterium tuberculosis***. *The Lancet* 1993, **341**(8846):647–651.

[120] Sreevatsan S, Pan X, Stockbauer KE, Williams DL, Kreiswirth BN, Musser JM: **Characterization of *rpsL* and *rrs***

mutations in streptomycin-resistant *Mycobacterium tuberculosis* isolates from diverse geographic localities. *Antimicrob. Agents Chemother.* 1996, **40**(4):1024–1026.

[121] Parish T, Stoker NG: **glnE is an essential gene in *Mycobacterium tuberculosis***. *Journal of Bacteriology* 2000, **182**(20):5715–5720.

[122] Zahrt TC, Deretic V: **An essential two-component signal transduction system in *Mycobacterium tuberculosis***. *Journal of Bacteriology* 2000, **182**(13):3832–3838.

[123] Saini DK, Tyagi JS: **High-throughput microplate phosphorylation assays based on DevR-DevS/Rv2027c 2-component signal transduction pathway to screen for novel antitubercular compounds**. *J Biomol Screen* 2005, **10**(3):215–224.

[124] Boon C, Dick T: ***Mycobacterium bovis* BCG response regulator essential for hypoxic dormancy**. *Journal of Bacteriology* 2002, **184**(24):6760–6767.

[125] Saini DK, Malhotra V, Dey D, Pant N, Das TK, Tyagi JS: **DevR–DevS is a bona fide two-component system of *Mycobacterium tuberculosis* that is hypoxia-responsive in the absence of the DNA-binding domain of DevR**. *Microbiology* 2004, **150**(Pt 4):865–875.

[126] Wehenkel A, Bellinzoni M, Graña M, Duran R, Villarino A, Fernandez P, Andre-Leroux G, England P, Takiff H, Cerveñansky C, Cole ST, Alzari PM: **Mycobacterial Ser/Thr protein kinases and phosphatases: physiological roles and therapeutic potential.** *Biochim Biophys Acta* 2008, **1784**:193–202.

[127] Scherr N, Honnappa S, Kunz G, Mueller P, Jayachandran R, Winkler F, Pieters J, Steinmetz MO: **Structural basis for the specific inhibition of protein kinase G, a virulence factor of *Mycobacterium tuberculosis***. *Proc Natl Acad Sci U S A* 2007, **104**(29):12151–12156.

[128] Soellner MB, Rawls KA, Grundner C, Alber T, Ellman JA: **Fragment-based substrate activity screening method for the identification of potent inhibitors of the *Mycobacterium tuberculosis phosphatase* PtpB**. *J Am Chem Soc* 2007, **129**(31):9613–9615.

[129] Qiao C, Gupte A, Boshoff HI, Wilson DJ, Bennett EM, Somu RV, Barry III CE, Aldrich CC: **5'-O-[(N-Acyl)sulfamoyl]adenosines as Antitubercular Agents that Inhibit MbtA: An Adenylation Enzyme Required for Siderophore Biosynthesis of the Mycobactins**. *Journal of Medicinal Chemistry* 2007, **50**(24):6080–6094.

[130] Rodriguez GM, Voskuil MI, Gold B, Schoolnik GK, Smith I: ***ideR*, an essential gene in *Mycobacterium tuberculosis*: Role of IdeR in iron-dependent gene expression, iron metabolism, and oxidative stress response**. *Infection and Immunity* 2002, **70**(7):3371–3381.

[131] Monfeli RR, Beeson C: **Targeting iron acquisition by *Mycobacterium tuberculosis***. *Infect Disord Drug Targets* 2007, **7**(3):213–220.
